# X-VAD: Video Anomaly Detection with Kinematic Explanations

**DOI:** 10.3390/s26216731

**Published:** 2026-10-24

**Authors:** Melisa Gözet, Asım Egemen Yılmaz, Mehmet Karaköse

**Affiliations:** 1 Department of Artificial Intelligence, Graduate School of Natural and Applied Sciences, Ankara University, 06110 Ankara, Türkiye; 2 Department of Artificial Intelligence Engineering, Faculty of Engineering, OSTİM Technical University, 06374 Ankara, Türkiye; 3 Department of Electrical and Electronics Engineering, Faculty of Engineering, Ankara University, 06830 Ankara, Türkiye; aeyilmaz@eng.ankara.edu.tr; 4 Department of Computer Engineering, Fırat University, 23119 Elazığ, Türkiye; mkarakose@firat.edu.tr

**Keywords:** video anomaly detection, video surveillance, optical flow, motion analysis, explainable artificial intelligence

## Abstract

Anomaly scores alone do not describe unusual motion. X-VAD links scoring and reporting through 13 cell-level kinematic descriptors from SEA-RAFT optical flow. They describe speed, direction, flow deformation, and temporal change. Each vector is compared with normal training observations from the same cell. The four highest calibrated cell scores are averaged to obtain the frame score. No gradient-based training of an anomaly scorer is required. Rule-based alarm reports identify the region, descriptor deviations, and nearest normal reference. YOLO-World adds object context without changing the score. Micro-averaged area under the receiver operating characteristic curve (AUROC) reaches 85.78%, 84.42%, and 75.43% on UCSD Ped2, CUHK Avenue, and IITB-Corridor, respectively. At the 0.85 threshold, all stored test scores yield true-positive rates of 87.69%, 90.78%, and 62.73%. Corresponding false-positive rates are 41.46%, 46.32%, and 26.53%. Runtime measurements use an NVIDIA A100-SXM4-40GB at batch size 1. Mean score-availability times are 50.0, 50.6, and 82.6 ms per frame pair, excluding frame reading. Report generation, including YOLO-World inference, adds 16.3–18.3 ms per alarm frame on average. Visualization and notification delivery are excluded. The IITB-Corridor AUROC approaches VideoPatchCore’s 76.4%.X-VAD provides inspectable motion reports, although its AUROCs trail SETJiP on Ped2 (99.3%) and AI-VAD on Avenue (93.7%).

## 1. Introduction

Surveillance cameras have become a standard part of public safety infrastructure in places such as train stations, shopping centers, and university campuses [1,2]. Falling hardware costs continue to increase the number of installed cameras [3]. The resulting video streams are long and mostly uneventful. Normal activity is repetitive and predictable, whereas security-relevant events are rare, brief, and varied. Continuous manual monitoring of such footage is tedious and error-prone [4], which motivates automatic video anomaly detection (VAD). In VAD, an anomaly is an event that does not conform to the expected behavior of a scene [5]. The task is to learn what normal activity looks like and flag observations that depart from it.

Anomalous events are unbounded in type and rare in practice, so they cannot be represented by a predefined set of classes. Collecting representative examples of every anomaly for training is therefore not feasible [6,7]. VAD is commonly formulated as a normal-only problem, in which a model of normal activity is fitted exclusively to normal training videos. Test frames are scored by their deviation from this reference [6,7]. In this setting, the reference is tied to the scene in which it was learned. As Cao et al. [6] observe, an event may be normal in one scene and abnormal in another. Playing football, for example, is ordinary on a playground but anomalous on a road. A normal reference built for one camera view may therefore not transfer reliably to another.

Location also matters within a single scene. Georgescu et al. [7] give the example of a person crossing a road. This crossing is unremarkable on a crosswalk but can be considered anomalous elsewhere. The same context dependence applies to motion in campus surveillance. A single camera may cover walkways, building entrances, corridors, and open areas. A motion direction, speed, or activity level may be expected in one image region but unusual in another. Whether a motion pattern is anomalous therefore depends on both how and where the motion occurs. A VAD system for such scenes should represent region-specific patterns of normal motion as well as scene-level regularities. UCSD Ped2 [8], CUHK Avenue [9], and IITB-Corridor [10] provide fixed-camera benchmarks of pedestrian and corridor scenes in which these effects can be studied.

A frame-level anomaly score indicates the degree of departure but provides limited information about the motion associated with an alarm. This limitation matters in closed-circuit television (CCTV) footage. Occlusion, illumination changes, and cluttered backgrounds complicate the interpretation of an increased score [11]. Camera movement can also produce apparent background motion. The Avenue dataset, for example, includes a test sequence affected by camera shake. An operator who receives an alarm must decide whether it requires attention. A score alone does not show where in the image the departure occurred or what kind of motion produced it. It also does not show what the corresponding normal activity looks like. A useful report should therefore link the score to an observable image region and identifiable motion characteristics. These characteristics should be expressed in quantities that can be checked against the video itself.

Optical flow provides a dense description of apparent image motion [12] and has been used as the basis for motion-based anomaly detection. Early methods modeled activity at fixed locations, used space-time graphical models, or summarized flow orientations into histograms [13,14,15]. Their statistics are directly related to the observed motion field. Learned prediction, reconstruction, and memory methods subsequently provided alternative representations of normality [16,17,18]. Recent normal-only approaches combine complementary cues or learning objectives. Examples include optical flow with red–green–blue (RGB) appearance in a generative framework [19], frame prediction with adaptive feature clustering [20], transformer-based reconstruction [21], and spatial and temporal jigsaw objectives [22]. Others use pose sequences with hyperbolic graph embeddings [23], patch-level normality memories [24], or synthetic anomaly supervision generated from normal videos [25]. These approaches provide frame-level discrimination on standard benchmarks. However, their anomaly decisions generally rely on learned representations, prediction or reconstruction errors, memory distances, or related latent quantities. These quantities are not expressed as explicit local motion descriptors such as speed, direction, expansion, rotation, or shear.

A separate line of work addresses explanation directly. EVAL [26] stores location-dependent normal exemplars with interpretable appearance and motion attributes. Scene-relationship models interpret unusual events through object interactions [27]. Language-based approaches incorporate visual descriptions and language-model reasoning into scoring [28]. EVAL illustrates the value of pairing a deviation with a reference from the same spatial region. This pairing allows an alarm to be interpreted against the normal activity observed at that location.

This study asks whether explicit local measurements of image motion can provide an anomaly score and an inspectable description of the measurements underlying that score. The proposed method uses the same measurements for both purposes, without an additional learned anomaly-scoring model. X-VAD represents each cell of a fixed image grid using 13 kinematic descriptors, collectively termed H13. These descriptors are computed in the image plane from dense optical flow estimated by SEA-RAFT [29]. They summarize speed, direction, local expansion, rotation, shear, and temporal changes in the direction distribution within each cell. A scene-specific normal memory is built for every cell exclusively from normal training videos. Each test descriptor vector is compared with normal examples from the same cell using nearest-neighbor matching. This nearest-neighbor matching is similar to that used in patch-based memory approaches to image anomaly detection [30]. The largest departures across cells are aggregated into a nonparametric frame score.

**Figure 1:**
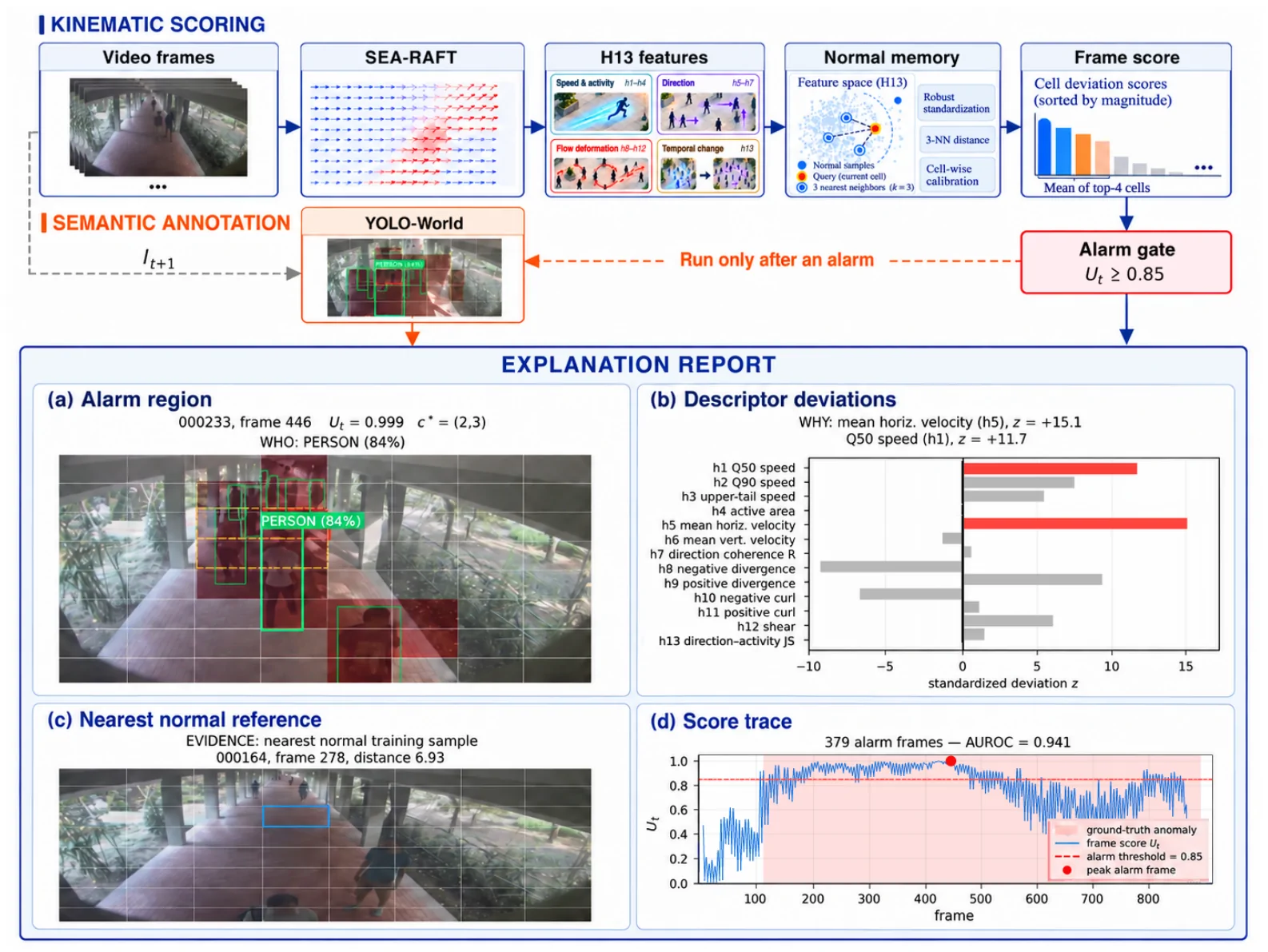
Overview of X-VAD. The top row shows kinematic scoring, and the middle row shows post-alarm semantic annotation. The bottom row shows the explanation report for IITB-Corridor video 000233, frame 446. Panel (**c**) shows the nearest normal training sample from training video 000164, frame 278.

Following an alarm, YOLO-World [31] supplies an associated object candidate. A rule-based report presents the alarm frame and selected region. It also presents the motion descriptors that deviate most from the normal medians and the nearest normal reference sample. The semantic branch operates after the kinematic decision and leaves the score unchanged. Detection therefore depends only on the measured motion descriptors, and the report can be checked against the frames it describes. Detection and kinematic interpretation are thus linked through the same H13 representation. The report describes deviations in the motion descriptors used for scoring, without requiring a separate learned model for kinematic explanation. Figure 1 gives an overview of the two branches.

The contributions of this study are summarized as follows:

- We propose a cell-level H13 representation that combines speed, direction, local flow deformation, and temporal distribution change. It uses normal references specific to each image region to express departures through explicit image-motion descriptors rather than only through latent learned representations.
- We design a scoring and reporting architecture that links kinematic departures to an alarm frame, image region, descriptor profile, and nearest normal reference. Post-alarm object annotation is kept separate from the score.
- We evaluate the framework on three fixed-camera benchmarks with a single configuration. Performance is measured using the frame-level micro-averaged AUROC. We also conduct an ablation study and analyze representative detections and false alarms to identify the limitations of motion-only normal references.

## 2. Related Work

Normal-only VAD methods differ in how they represent normal activity and what their output conveys about an alarm. Following recent surveys and benchmarks [1,6,11], we consider three lines of work. Motion-statistics models describe scene activity through explicit measurements of the image-motion field. Learned normality models score deviations through reconstruction, prediction, self-supervised proxy tasks, or stored prototypes. Explanation-oriented methods attach interpretable attributes, object relations, or language to the detection. Approaches that also use anomalous training videos, such as TDS-Net [2], address a different data setting and are not considered further. Section 2.1 and Section 2.2 review the first two lines, and Section 2.3 reviews the third.

### 2.1. Motion-Statistics Representations of Normal Activity

Early VAD methods modeled normal activity through statistics of the observed motion field. Adam et al. [13] placed fixed-location monitors in the image and learned the distribution of local flow observations at each monitor. An alarm was raised when the motion at a location became improbable. Kim and Grauman [14] used a space-time Markov random field over local regions. Their model assessed each region together with its spatial and temporal neighbors and supported incremental updates. Wang and Snoussi [15] summarized optical flow into orientation histograms and used one-class classifiers trained on normal frames. These methods use explicit flow measurements, and the fixed-location models retain spatial context. X-VAD follows this motion-based formulation and combines speed and direction with local deformation and temporal change. It also reports descriptor deviations and retrieves a normal example from the same image cell.

### 2.2. Learned Normality Models

Learned generative models assess anomalies through differences between observed frames and frames reconstructed or predicted by a network trained on normal data. Liu et al. [16] used future-frame prediction error as the anomaly score. MemAE [17] and MNAD [18] added memory modules that store normal prototypes and constrain reconstruction or prediction. Transformer-based variants use a video vision transformer for frame prediction [5] or jointly predict future frames and optical-flow magnitudes with adversarial training [4]. Other work combines transformers with ensembles of convolutional autoencoders [3], and a recent review surveys this direction [1]. Multi-cue and multi-objective designs extend these approaches. Kang and Kang [19] integrate optical flow and RGB appearance in a generative adversarial framework. MS-UACL [20] combines frame prediction with adaptive feature clustering. ViT-STRA [21] pairs a vision transformer with spatiotemporal relationship attention for reconstruction. These studies report normal-only evaluations on at least one of Ped2 and Avenue.

Self-supervised proxy tasks provide an alternative to generative objectives. Georgescu et al. [7] train an object-centric network on several tasks, including arrow-of-time and motion-irregularity prediction, and score objects using task errors. SETJiP [22] learns spatiotemporal structure through spatial and temporal jigsaw puzzles and strengthens the temporal objective for global-motion examples. HGNF [23] models human-pose sequences using hyperbolic graph embeddings and normalizing flows, with a normal-only evaluation on IITB-Corridor. VideoPatchCore [24] stores spatial, temporal, and high-level patch features of normal videos in memory banks. It scores test patches by their distance to stored features without training an anomaly scorer. This nearest-neighbor principle is also used for image anomaly detection with frozen foundation-model features [30]. Wang et al. [25] generate synthetic anomalous videos from normal ones to obtain fine-grained supervision. Cao et al. [6] introduced the NWPU Campus benchmark to study events whose normality depends on the scene. This setting extends the evaluation beyond single-scene datasets.

Across these models, anomaly decisions rely on learned embeddings, prediction or reconstruction residuals, or distances in learned feature spaces. These quantities support frame-level discrimination but do not directly describe local motion in kinematic terms. X-VAD shares the memory-based scoring principle of VideoPatchCore [24] and AnomalyDINO [30], which do not train an anomaly scorer by gradient descent. Its memory stores explicit flow-derived descriptors for each image cell. The same representation therefore supports scoring as well as the reporting of descriptor deviations and a nearest normal example.

### 2.3. Explainable and Location-Dependent Detection

EVAL [26] is the closest methodological reference for this study. It learns appearance and motion representations from video volumes and stores representative exemplars separately for each spatial region. A test volume is compared with exemplars from its own region. Predicted attributes, such as object class and motion direction, describe the volume’s content. X-VAD also uses region-specific normal references, but its 13 descriptors are computed directly from optical flow. Appearance information is added only after an alarm, so the scoring distance depends on motion alone.

Other explanation strategies use object relations or language. Doshi and Yilmaz [27] describe frames through scene graphs and interpret unusual events through object interactions. LAVAD [28] generates frame descriptions with a vision-language model and scores anomalies through language-model reasoning without task-specific training. X-VAD instead describes departures from local normal motion through fixed reporting rules and a retrieved normal example. Region- and track-based protocols developed for Street Scene [32] address the separate question of spatial localization accuracy. The present evaluation covers frame-level discrimination and qualitative inspection of the reported regions.

These studies provide motion statistics, learned representations, and several forms of explanation. X-VAD combines location-specific normal memories with explicit kinematic descriptors and requires no gradient-based training of an anomaly scorer. The descriptors form the scoring representation and supply the motion measurements shown in each report.

## 3. Problem Formulation

A scene is a fixed camera view with normal training videos and test videos. A video of *T* frames $I_{1},\ldots ,I_{T}$ yields T−1 consecutive pairs $\left(I_{t},I_{t+1}\right)$, t=1,…,T−1. Pairs do not cross video boundaries. X-VAD assigns a score $U_{t}\in \left[0,1\right]$ to each pair and attributes it to the later frame $I_{t+1}$. The temporal descriptor also uses motion history from the same video. Each video therefore receives T−1 frame scores, and its first frame is unscored.

Processed frames have width *W*, height *H*, and diagonal $D=\sqrt{W^{2}+H^{2}}$ pixels. The frame rate is fps (frames per second), and the frame interval is δt=1/fps. The image is divided into an 8×8 grid of $N_{C}=64$ cells. Each cell contains $N_{P}=\left(H/8\right)\left(W/8\right)$ pixels and is indexed by c∈{1,…,64}.

Let $V_{\mathrm{tr}}$ and $V_{\mathrm{te}}$ denote the normal training videos and test videos of a scene. The task is to fit the scene-specific reference using only $V_{\mathrm{tr}}$ and score the pairs in $V_{\mathrm{te}}$. Anomalous frames ($y_{t+1}=1$) should receive higher scores than normal frames ($y_{t+1}=0$). AUROC evaluates this ranking (Section 5.2). Test labels do not enter fitting, scoring, or report generation. They are used for evaluation and overlaid on report traces only for illustration. Whenever $U_{t}\geq \theta$, the method also returns the explanation tuple

(1) $$E_{t}=\left(t+1,\;c^{*},\;T_{t},\;{\left\{\left(j,z_{t,c^{*},j}\right)\right\}}_{j\in J_{t}},\;r_{t},\;o_{t}\right).$$

The tuple contains the alarm frame, highest-scoring cell, selected cell set, and two descriptors with the largest absolute standardized deviations from the cell’s normal medians. It also includes their signed standardized values, the nearest normal sample $r_{t}\in M_{c^{*}}$, and an optional object candidate $o_{t}$. The object candidate does not enter $U_{t}$. Scoring and kinematic reporting use the same H13 representation, while object annotation remains separate. Table 1 lists the main symbols used in Section 3 and Section 4.

## 4. Proposed Method: X-VAD

Figure 1 shows the two branches of X-VAD. The kinematic branch estimates dense optical flow and summarizes each cell of a fixed 8×8 image grid using 13 descriptors. It compares each descriptor vector with a cell-specific normal memory and aggregates the largest deviations into a frame score. When the score reaches the alarm threshold, the semantic branch applies an open-vocabulary object detector to the alarm frame. A rule-based report combines the motion measurements with the associated object candidate.

Active-motion thresholds, descriptor-scaling statistics, normal memories, and calibration distributions are fitted exclusively to the normal training videos of each scene. SEA-RAFT and YOLO-World use released weights pretrained on external data and are not fine-tuned. Section 4.1, Section 4.2, Section 4.3 and Section 4.4 describe optical flow, H13, scoring, and reporting. Section 5 gives the datasets, evaluation protocol, and implementation details.

### 4.1. Dense Optical Flow Estimation

Optical flow describes the apparent displacement of image content between two frames [12]. SEA-RAFT builds on recurrent all-pairs field transforms (RAFT), which iteratively refine flow using correlations between image features [29]. It predicts an initial flow before refinement and uses a mixture-of-Laplace training loss and rigid-motion pretraining. Its published results report strong accuracy and cross-dataset generalization at a lower computational cost than comparably accurate methods. These properties, together with the released code and weights [33,34], motivated its use in X-VAD. Dense flow supplies measurements for every image cell without first detecting or tracking objects. Flow quality matters because speed depends on displacement estimates, while deformation descriptors also depend on their spatial derivatives.

X-VAD uses SEA-RAFT [29] with the released spring-M model and checkpoint [33,34]. The model runs for four refinement iterations. The input scale is set to 0, so flow is estimated at the processed image resolution rather than the half-resolution setting of the released evaluation configuration. Each processed dimension must be a multiple of 8. The three image sizes in Section 5.1 satisfy this requirement without cropping.

For a frame pair $\left(I_{t},I_{t+1}\right)$, the raw flow $F_{t}$ in pixels is normalized by the image diagonal and the frame interval,

(2) $$f_{t}=\frac{F_{t}}{D\;\delta t},\;m_{t}={∥f_{t}∥}_{2}.$$

Speed is therefore expressed in the common image-relative unit of image diagonals per second. A Gaussian-smoothed field ${\bar{f}}_{t}=G_{\sigma }*f_{t}=\left({\bar{f}}_{u},{\bar{f}}_{v}\right)$ with σ=0.002D is used for spatial derivatives. With *x* and *y* denoting pixel coordinates,

(3) $$J_{11}=D\;\partial_{x}{\bar{f}}_{u},\;J_{12}=D\;\partial_{y}{\bar{f}}_{u},\;J_{21}=D\;\partial_{x}{\bar{f}}_{v},\;J_{22}=D\;\partial_{y}{\bar{f}}_{v},$$

and the local deformation fields are

(4) $$\mathrm{div}=J_{11}+J_{22},\;\mathrm{curl}=J_{21}-J_{12},\;\mathrm{shear}=\sqrt{{\left(J_{11}-J_{22}\right)}^{2}+{\left(J_{12}+J_{21}\right)}^{2}}.$$

They measure local expansion or contraction, signed rotation, and shear of the image-motion field.

### 4.2. H13 Kinematic Descriptors

#### 4.2.1. Active-Motion Threshold

A cell-specific threshold defines the active pixels used to construct the descriptor supports. For the normalized magnitudes ${\tilde{m}}_{c}$ of cell *c* observed in the normal training videos,

(5) $$\tau_{c}=\max\;\left\{\mathrm{median}\left({\tilde{m}}_{c}\right)+3\;\left(1.4826\right)\;\mathrm{MAD}\left({\tilde{m}}_{c}\right),\;\frac{0.3}{D\;\delta t}\right\},$$

where MAD is the median absolute deviation. The factor 1.4826 makes the scaled MAD comparable to a standard deviation under normality [35]. The second term is a lower bound of 0.3 pixels per frame, expressed in the units of Equation (2). Threshold statistics use a deterministic subsample of training pairs, with 16 pixels sampled per cell in each selected pair. A fixed pair stride targets approximately 5000 pairs. This stride gives 2534 pairs for Ped2, 5111 for Avenue, and 5128 for IITB-Corridor. Appendix A.2 gives the stride, and Appendix A.6 gives the pixel indices. In all three scenes, the MAD-based term falls below the lower bound in every cell. Each scene therefore uses the constant threshold listed in Section 5.1. The active set is $A=\{m>\tau_{c}\}$. Its dilated support $A^{+}$ is obtained using a square maximum filter with half-width ⌈σ⌉. The filter is applied to the whole-image active mask before restriction to each cell. The resulting support is used for the deformation descriptors.

#### 4.2.2. Speed, Direction, and Deformation Descriptors

Table 2 defines the 13 descriptors. Descriptors $h_{1}$–$h_{7}$ use the active set *A* to summarize speed, motion extent, mean direction, and direction coherence. Descriptors $h_{8}$–$h_{12}$ use $A^{+}$ to summarize the tails of the deformation fields in Equation (4). For a support of *N* pixels, the lower and upper tail means average the smallest and largest min{max(⌈0.01N⌉,8),N} values, respectively. They retain extreme motion measurements that an average alone would not describe. The speed and direction group requires |A|≥8, defining a *motion-valid* cell. The deformation group requires $|A^{+}|\geq 8$. Invalid descriptors are set to zero, except that the raw active fraction is retained for the temporal distribution. When the cell is not motion-valid, the standardized coordinate of this fraction is set to zero.

#### 4.2.3. Temporal Descriptor

The temporal descriptor compares the direction–activity distribution of a cell with that of the same cell Δ pairs earlier. The gap Δ=max{1,round(0.2fps)} corresponds to approximately 0.2 s at the frame rate used (Section 5.1). Active flow vectors form an eight-bin circular direction histogram $H_{t}$. Each vector is weighted by its magnitude, with the weight distributed linearly between adjacent bins. The normalized histogram is combined with the inactive fraction into a nine-component distribution,

(6) $$P_{t}=\left[\;1-h_{4,t},\;h_{4,t}H_{t,1},\ldots ,h_{4,t}H_{t,8}\;\right].$$

The descriptor $h_{13}$ is the Jensen–Shannon (JS) divergence [36], normalized to [0,1],

(7) $$h_{13,t}=\frac{\frac{1}{2}\mathrm{KL}\left(P_{t}\;∥\;M\right)+\frac{1}{2}\mathrm{KL}\left(P_{t-\Delta }\;∥\;M\right)}{\log2},\;M=\frac{1}{2}\left(P_{t}+P_{t-\Delta }\right),$$

where KL(·∥·) denotes the Kullback–Leibler divergence. The descriptor is valid for t>Δ and is set to zero for the first Δ pairs of a video. Numerical stabilization of the logarithms and the complete validity rules are given in Appendix A.2.

### 4.3. Cell-Specific Normal Memory and Frame Score

#### 4.3.1. Robust Standardization

Normality is modeled separately for each cell. For every cell *c* and descriptor *j*, the median $\mu_{c,j}$ and scaled MAD are computed from valid training entries. At least ten entries are required. Otherwise, both statistics are set to zero. A scene-level floor ε prevents division by near-zero scales. It is the 10th percentile of all positive scaled MADs in the scene, with a fallback of $10^{-6}$ if none is positive. The final scale is ${\hat{s}}_{c,j}=\max\left\{1.4826\;{\mathrm{MAD}}_{c,j},\varepsilon \right\}$, giving

(8) $$z_{t,c,j}=\frac{h_{t,c,j}-\mu_{c,j}}{{\hat{s}}_{c,j}}.$$

Invalid descriptors are set to zero after standardization.

#### 4.3.2. Memory Construction and Out-of-Fold Calibration

The memory $M_{c}$ contains standardized vectors from motion-valid training observations of cell *c*. At least 100 such observations are required. If more than 40,000 are available, a deterministic, evenly spaced subsample of 40,000 vectors is retained. Scoring also requires at least one calibration distance, as specified in Appendix A.3. For a query vector *z*, the cell distance is the mean Euclidean distance to its k=3 nearest neighbors in the memory,

(9) $$d_{c}\left(z\right)=\frac{1}{k}\sum_{\ell =1}^{k}{∥z-z_{c}^{\left(\ell \right)}∥}_{2},\;k=3,$$

computed exactly by exhaustive search.

A self-match contributes a zero distance and lowers the mean nearest-neighbor distance. Normal reference distances are therefore obtained by video-grouped four-fold calibration. Training videos are assigned to folds by their index modulo four. Vectors from each fold are queried against a memory built from the other three folds. This scheme excludes both the query and other observations from its video. The resulting out-of-fold distances are pooled per cell and sorted, giving the reference set ${\left\{d_{i}^{\left(c\right)}\right\}}_{i=1}^{n_{c}}$. At test time, the query is compared with the full memory, and its distance is mapped to an empirical percentile,

(10) $$p_{t,c}=\frac{1+\#\{i:\;d_{i}^{\left(c\right)}<d_{t,c}\}}{n_{c}+1}.$$

Nonscorable cells and cells that are not motion-valid at pair *t* receive $p_{t,c}=0$. The percentile is an empirical distance reference rather than a conformal probability or a prescribed false-alarm rate. The reference distances are computed against memories built from three of the four folds, whereas test queries are compared with the full memory. The thresholds and scaling statistics are fitted once on the entire training split.

#### 4.3.3. Frame Score and Alarm

Let $T_{t}$ denote the set of the four cells with the largest $p_{t,c}$ (the top $\lceil 0.05N_{C}\rceil =4$ cells). The frame score is the mean of these four percentiles,

(11) $$U_{t}=\frac{1}{4}\sum_{c\in T_{t}}p_{t,c},\;\left|T_{t}\right|=\left\lceil 0.05\times 64\right\rceil =4,$$

which emphasizes regions with the largest departures from local normal motion. Nonscorable and motion-invalid cells contribute zero, while the denominator remains four. Four nonzero cell responses are therefore required for the score to reach its maximum. The main evaluation uses this score without temporal smoothing, per-video normalization, or threshold tuning. An alarm is raised when $U_{t}\geq \theta$, with θ=0.85. This threshold controls report generation, whereas AUROC evaluates the continuous score over all thresholds. Algorithm 1 summarizes fitting and scoring, and Table 3 gives the resulting memory statistics.
**Algorithm 1** X-VAD kinematic scoring for one scene**Require:** Normal training videos $V_{\mathrm{tr}}$; test video *V*; constants σ=0.002D, Δ, k=3, θ=0.85**Ensure:** Frame scores $U_{t}$ and alarm set $\left\{t:U_{t}\geq \theta \right\}$*   Normal-only fitting*  1: Estimate $\tau_{c}$ for every cell from a deterministic subsample of training pairs▹ Equation (5)  2: **for all** training pairs $\left(I_{t},I_{t+1}\right)$ in $V_{\mathrm{tr}}$ **do**  3:      $F_{t}\leftarrow$ SEA-RAFT$\left(I_{t},I_{t+1}\right)$; $f_{t},m_{t}\leftarrow$ Equation (2)  4:      Compute $h_{t,c,1..13}$ and validity flags for all cells▹ Table 2, Equations (4)–(7)  5: **end for**  6: Compute $\mu_{c,j}$ and ${\hat{s}}_{c,j}$; standardize to $z_{t,c}$▹ Equation (8)  7: **for all** cells *c* with at least 100 motion-valid observations **do**  8:       $M_{c}\leftarrow$ motion-valid $z_{t,c}$ (evenly spaced subsample if >40,000)  9:       Compute out-of-fold distances $\left\{d_{i}^{\left(c\right)}\right\}$ by video-grouped four-fold *k*-NN▹ Equation (9)10: **end for**   *Test-time scoring*11: **for all** pairs $\left(I_{t},I_{t+1}\right)$ in *V* **do**12:       Compute $z_{t,c}$ for all cells with the training statistics13:       **for all** scorable cells *c* **do**14:             $p_{t,c}\leftarrow$ percentile of $d_{c}\left(z_{t,c}\right)$ among $\left\{d_{i}^{\left(c\right)}\right\}$▹ Equation (10)15:       **end for**16:       $p_{t,c}\leftarrow 0$ for nonscorable or motion-invalid cells17:       $U_{t}\leftarrow$ mean of the four largest $p_{t,c}$▹ Equation (11)18:       **if** $U_{t}\geq \theta$ **then**19:             Generate the explanation report for frame $I_{t+1}$
▹ Section 4.420:       **end if**21: **end for**


### 4.4. Post-Alarm Semantic Annotation and Explanation Report

For each alarm pair *t*, the report uses frame $I_{t+1}$, the highest-scoring cell $c^{*}=\arg\max_{c}p_{t,c}$, and the four cells in $T_{t}$. It presents three kinematic elements. First, it lists the two descriptors of $c^{*}$ with the largest absolute standardized values $|z_{t,c^{*},j}|$, retaining their signs. These values describe departures from the cell’s normal medians. Second, it retrieves the nearest normal vector of $c^{*}$ from the full memory using k=1. The report identifies its training video and frame and gives the Euclidean distance in standardized descriptor space. This retrieval provides an observed normal example at the same location. Third, it displays the video’s frame-score trace and alarm threshold. The displayed descriptor ranking describes marginal deviations and is not an additive decomposition of the k=3 scoring distance or frame score.

YOLO-World [31] is then applied to $I_{t+1}$ through the Ultralytics implementation [37]. It uses the released yolov8m-worldv2 weights, a confidence threshold of 0.25, and an input size of 640 pixels. Its vocabulary contains 31 prompts encoded by the text encoder of Contrastive Language–Image Pretraining (CLIP) [31,38]. These prompts name people and visible states, vehicles, weapons and tools, bags and containers, fire, smoke, and dogs (Appendix C). Motion concepts such as running and fighting are excluded because the kinematic branch handles motion. The box with the largest intersection area with $c^{*}$ supplies the associated object candidate, class, and confidence. If no box intersects the cell, the report states that no candidate was associated and retains the kinematic alarm. YOLO-World runs only for alarm frames and never modifies $U_{t}$.

Fixed templates assemble these elements into a report. The report states when the alarm occurred (video and frame) and where the strongest response was located (cell $c^{*}$ and $T_{t}$). It also lists the motion measurements that departed most from the normal medians and their direction, the nearest normal training sample in H13 space, and the associated object candidate, if any. Figure 1 (bottom row) shows one report. Panel (a) shows the alarm frame with the percentile heat map, the highest-scoring cell $c^{*}$ (red), and the remaining cells of $T_{t}$ (dashed orange). Thin green boxes show detections, while a thicker labeled box identifies the associated candidate, if any. Panel (b) shows the standardized H13 values of $c^{*}$, with the two largest departures highlighted. Panel (c) shows the nearest normal training sample of $c^{*}$ with its frame index and descriptor-space distance. Panel (d) is the frame-score trace with the ground-truth anomaly interval (shaded), the alarm threshold (dotted), and the peak alarm frame (red dot). Additional complete reports are given in Appendix D.

## 5. Experiments

### 5.1. Datasets and Preprocessing

Three publicly available fixed-camera benchmarks are used. Each is treated as a separate scene with its own normal reference.

- **UCSD Ped2** [8]: 16 training videos (2550 frames) and 12 test videos (2010 frames) of a pedestrian walkway at 360×240 pixels. Anomalies are nonpedestrian entities, such as cyclists, skaters, and small vehicles, on the walkway.
- **CUHK Avenue** [9]: 16 training videos (15,328 frames) and 21 test videos (15,324 frames) at 640×360 pixels. Anomalies include running, throwing objects, and loitering. The dataset documentation notes a small number of outliers in the training split and a test sequence affected by camera shake. The training split is used as released.
- **IITB-Corridor** [10]: 208 training videos (301,999 frames) and 150 test videos (181,567 frames) of a campus corridor recorded at 1920×1080 pixels. Anomalies include fighting, chasing, loitering, and unattended baggage.

Ped2 and Avenue frames are processed at their native resolution. IITB-Corridor frames are resized to 1024×576 pixels with area interpolation, a leading black frame is removed when present, and the annotations are aligned with the retained sequence. The frame counts above are given after this removal. Frame rates are taken as 10 fps for Ped2, whose frame sequences carry no timing information, and as the decoded rate of 25 fps for Avenue. For IITB-Corridor, whose videos are reported at 25 fps [39], the frames were processed as an extracted image sequence with an assumed rate of 30 fps. Section 5.7 shows that using 25 fps instead changes the micro AUROC by 0.03 points. Table 4 lists the resulting geometry. The scene-dependent quantities in its lower part follow from *D* and δt through the definitions given in Section 4.1 and Section 4.2. Complete preprocessing and label-alignment rules are given in Appendix A.1.

### 5.2. Evaluation Protocol

In line with most VAD studies [16,17,18,22,24,26], detection performance is measured by frame-level AUROC [40]. Let P and N denote the anomalous and normal subsets of the scored frames, respectively. AUROC measures how often an anomalous frame receives a higher score than a normal frame, with half credit for tied scores,

(12) $$\mathrm{AUROC}\left(P,N\right)=\frac{1}{\left|P|\;|N\right|}\sum_{i\in P}\sum_{i^{\prime }\in N}\left[1\left(U_{i}>U_{i^{\prime }}\right)+\frac{1}{2}\;1\left(U_{i}=U_{i^{\prime }}\right)\right].$$

Here, *i* and $i^{\prime }$ index scored frames in the evaluation set, $U_{i}$ denotes the score assigned to frame *i*, and 1(·) is the indicator function. Each $U_{i}$ is the pair score ending at that frame. Micro-averaged AUROC pools frames from all annotated test videos, with $P={⋃}_{v}P_{v}$ and $N={⋃}_{v}N_{v}$. Macro-averaged AUROC is $|V^{\pm }{|}^{-1}\sum_{v\in V^{\pm }}\mathrm{AUROC}\left(P_{v},N_{v}\right)$, where $V^{\pm }$ contains only videos with both labels. The pooled micro average [24,41] also includes videos containing only normal or only anomalous frames. Each video’s first frame is excluded because it has no score. The main results use unsmoothed scores without per-video normalization. Alarm behavior is summarized by the false-positive rate (FPR) and true-positive rate (TPR) at θ=0.85, computed from the stored scores of all scored test frames. FPR is the fraction of normal frames reaching the threshold, and TPR is the corresponding fraction of anomalous frames. Section 5.7 evaluates how smoothing changes both rates.

### 5.3. Implementation Details and Computational Cost

The main configuration was fixed before test evaluation and used for all three datasets without test-based tuning.

We measured frame-by-frame runtime on a single NVIDIA A100-SXM4-40GB GPU at batch size 1 (Table 5). For each dataset, we timed the first 1000 test pairs after 50 warm-up pairs, in video order. Each frame was paired with the preceding frame from the same video. No future frames were used. Score-availability time starts before tensor conversion and GPU transfer of the current frame. It ends when the frame score is available, before thresholding. It includes optical flow, descriptor extraction, memory lookup, and scoring. Frame reading is measured separately and includes resizing for IITB-Corridor.

Report generation is timed only for alarm frames. It includes nearest-normal retrieval, YOLO-World inference and object association, and text-report assembly. Loading the normal reference image, rendering the report figure, and notification delivery are excluded.

Optical flow accounts for 81–84% of score-availability time. Report generation adds 16.3–18.3 ms per alarm frame on average. The score-availability rates are 20.0, 19.8, and 12.1 frames/s for Ped2, Avenue, and IITB-Corridor, respectively. These rates exceed the assumed 10 fps for Ped2 but remain below 25 fps for Avenue and IITB-Corridor. Including frame reading reduces the IITB-Corridor rate to approximately 10 frames/s before report generation. Peak GPU allocation is reported by PyTorch; host memory was not measured.

The main AUROC, TPR, and FPR results use stored offline scores. Online and offline alarm decisions agree for 99.7–99.9% of the timed pairs. Detailed consistency checks and the sampled video identifiers are provided in the appendix.

Appendix A.5 lists the software versions.

### 5.4. Frame-Level Anomaly Detection

Table 6 reports results under the same pooled evaluation protocol for all three datasets. X-VAD achieves micro AUROC values of 85.78% on Ped2, 84.42% on Avenue, and 75.43% on IITB-Corridor. The corresponding macro AUROCs, averaged over the videos that contain both normal and anomalous frames, are 89.22%, 86.29%, and 74.34%.

At θ=0.85, the TPRs are 87.69%, 90.78%, and 62.73%, and the FPRs are 41.46%, 46.32%, and 26.53%. These rates are computed from the stored scores of all scored test frames and describe the operating point used for report generation; Section 5.7 shows how causal smoothing changes them.

Appendix B provides the per-video results for Ped2 and Avenue.

### 5.5. Comparison with Published Methods

Table 7 compares X-VAD with published frame-level results, including the methods reviewed in Section 2. AI-VAD [42] provides an attribute-based reference that combines object velocity, pose, and pretrained deep features. The published results use their original evaluation protocols and were not rerun with common preprocessing or postprocessing. Table 7 therefore lists the scoring input and the postprocessing stated in each publication. These differences should be considered when interpreting the comparison. X-VAD uses motion descriptors for scoring and applies YOLO-World only after an alarm.

On IITB-Corridor, X-VAD achieves 75.43%, compared with 76.4% for VideoPatchCore, 72.4% for HGNF, and 67.12% for MTP. VideoPatchCore also avoids training an anomaly scorer but uses object regions and pretrained features. X-VAD reaches 84.42% on Avenue, compared with 93.7% for AI-VAD. On Ped2, it reaches 85.78%, while the highest listed result is 99.3%. Ped2 contains nonpedestrian anomalies, and only 24 of its 64 cells meet X-VAD’s memory requirements (Table 3). These findings motivate closer examination of the motion representation and spatial coverage. EVAL also evaluates region- and track-based localization. Its frame-level Avenue result is included here to match the reported evaluation metric.

### 5.6. Ablation Study

Table 8 evaluates descriptor groups and scoring choices on Ped2, Avenue, and IITB-Corridor. Each variant changes one component using stored descriptors. Distances and calibration are recomputed when the descriptor coordinates, *k*, or calibration setting changes. Aggregation variants use the same cell scores and change only the number of selected cells. The baseline reproduces the original cell and frame scores exactly. Descriptor ablations retain the full model’s standardization statistics and validity rules, so they isolate coordinate removal within the existing representation. All fitted quantities use normal training data, and no variant replaces the fixed main configuration.

The descriptor groups have different effects in the three scenes. Removing direction increases Ped2 AUROC by 4.33 percentage points but decreases Avenue AUROC by 2.62 points. Removing deformation decreases Ped2 AUROC by 2.32 points and increases Avenue AUROC by 1.30 points. Deformation removal causes the largest group-level decrease on Ped2, while direction removal causes the largest decrease on Avenue and IITB-Corridor (2.62 and 1.63 points, respectively). Removing temporal change decreases Avenue AUROC by 2.49 points but increases Ped2 and IITB-Corridor AUROC by 1.22 and 0.19 points, respectively. The results support complementary, scene-dependent roles for the groups, rather than a claim that every group improves performance on every dataset. The full H13 set is retained to avoid selecting dataset-specific subsets based on test performance.

Aggregation and calibration have the largest effects among the scoring choices. Top-1 decreases AUROC by 14.60 points on Ped2 and 1.43 points on IITB-Corridor, while top-8 decreases Avenue AUROC by 2.87 points. Top-4 gives the highest tested aggregation result on Ped2 and IITB-Corridor and is 0.60 points below top-2 on Avenue. Changing *k* from 3 to 1 or 5 changes AUROC by less than 0.5 points. Using two or eight calibration folds changes it by at most 0.14 points. The leave-self-out variant excludes only the query observation. Other observations from the same video remain eligible. Its changes are +0.35, −0.03, and −0.19 points on Ped2, Avenue, and IITB-Corridor, respectively. Thus, four-fold calibration is not uniquely optimal in these experiments. However, it retains a video-separated reference and prevents self-matches. Replacing calibrated percentiles with raw *k*-NN distances decreases AUROC by 15.30, 1.13, and 0.91 points on Ped2, Avenue, and IITB-Corridor, respectively, supporting per-cell calibration for the tested configuration.

Table 9 reports the removal of each descriptor separately. On Ped2, removing horizontal motion ($h_{5}$) causes the largest individual decrease, at 1.18 points. Removing direction coherence ($h_{7}$) instead increases AUROC by 3.42 points. On Avenue, temporal change ($h_{13}$) has the largest individual removal effect, at −2.49 points, followed by vertical motion ($h_{6}$), at −1.59 points. On IITB-Corridor, direction coherence ($h_{7}$) has the largest individual decrease, at 0.57 points, followed by horizontal motion ($h_{5}$), at 0.36 points. These results measure conditional contributions within H13, not independent feature importance or explanation faithfulness. They do not establish that all 13 descriptors are individually necessary.

### 5.7. Score Smoothing and Assumed Frame Rate

Table 10 evaluates causal moving averages of the frame scores. Each window contains the current score and preceding scores from the same video. At the start of a video, only the available history is used. Fitting remains unchanged.

A window of about 0.5 s increases micro AUROC by 1.99 points on Ped2 and 0.96 points on IITB-Corridor, but decreases it by 0.19 points on Avenue. At θ=0.85, FPR decreases from 41.46% to 36.13% on Ped2, from 46.32% to 44.56% on Avenue, and from 26.53% to 23.49% on IITB-Corridor. The corresponding TPR changes, relative to the unsmoothed values in Table 6, are +0.49, −1.19, and −1.39 points, respectively. Smoothing therefore reduces FPR at this threshold, with different effects on ranking and sensitivity across datasets. The main results retain unsmoothed scores to link each report directly to the target frame’s kinematic score. The temporal history used by $h_{13}$ remains part of that score.

The frame-rate experiment varies the assumed frame rate while retaining the same frame pairs. It evaluates the frame-rate coefficient in descriptor computation, rather than temporal subsampling. The assumed rate determines δt and the rounded temporal gap Δ. Changing δt rescales speed, deformation, and the active-motion threshold by the same factor. Per-cell standardization largely removes this common scaling. Remaining differences can arise from Δ, numerical effects, and the scale floor shared across descriptors.

For Ped2 and Avenue, a shared flow extraction is used across the frame-rate settings, and the thresholds are re-estimated for each rate. IITB-Corridor variants are derived from stored descriptors and direction–activity distributions. Their thresholds scale with the rate, so the active masks are unchanged. Scaling statistics, memories, and calibration are refitted on normal training data for each rate. Table A1 reports the results, with changes measured against the reference rate of each sensitivity run.

Across the reported rates, the largest absolute AUROC changes are 0.93 points on Ped2, 0.44 on Avenue, and 0.12 on IITB-Corridor. Using the reported 25 fps instead of the assumed 30 fps on IITB-Corridor changes AUROC from 75.43% to 75.40%. These results indicate limited sensitivity to the assumed frame-rate coefficient over the evaluated ranges.

### 5.8. Qualitative Explanation and Failure Analysis

**Figure 2:**
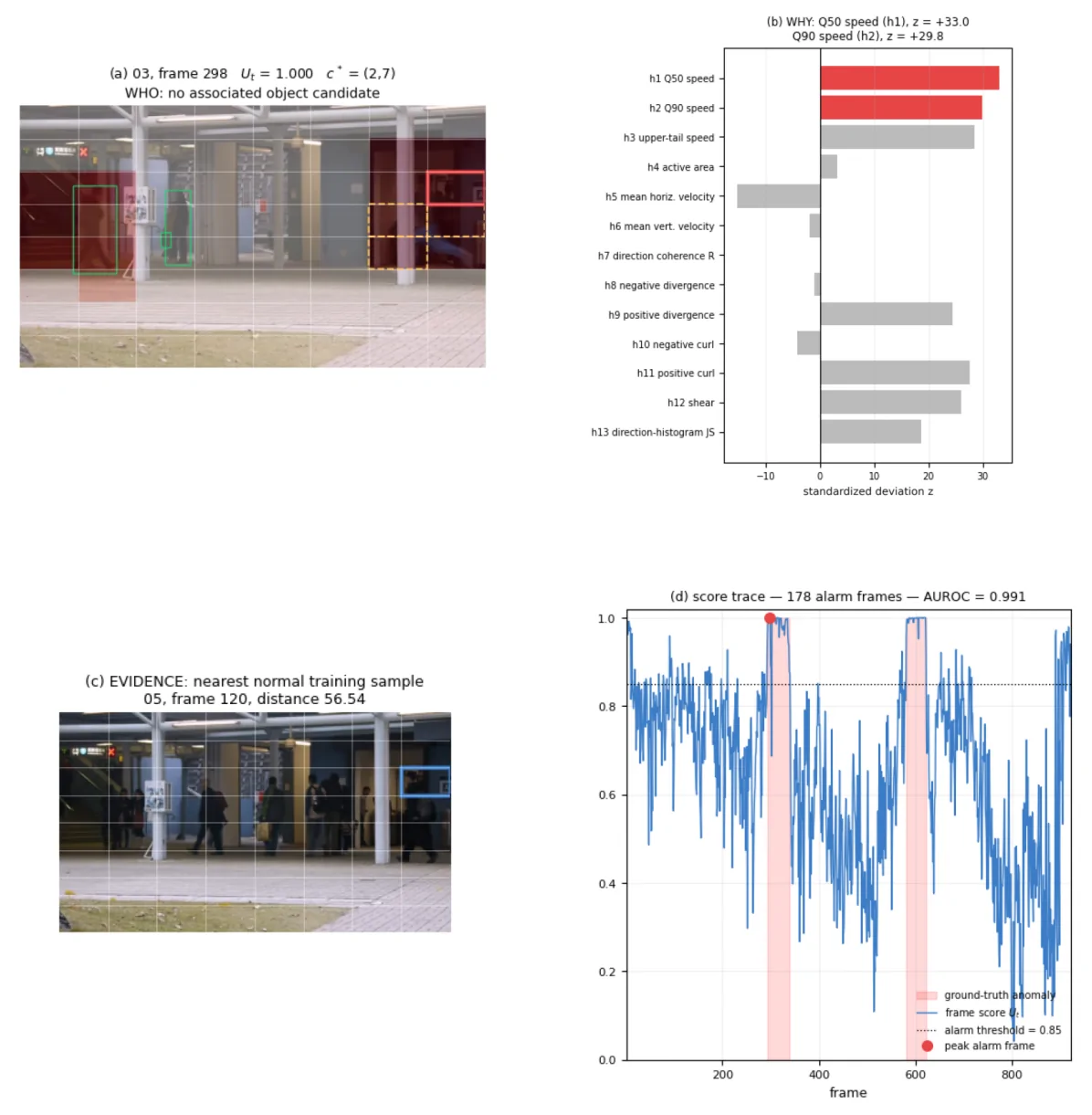
Avenue video 03: complete explanation report at frame 298 (within-video AUROC 99.1%).

The qualitative analysis considers a high-performing Avenue video and a false-alarm example from IITB-Corridor. Each displayed frame is the frame with the maximum score within its video (the first such frame in the case of ties). Figure 2 shows the complete explanation report for Avenue video 03, whose within-video AUROC is 99.1%. At frame 298, the report identifies elevated median speed ($h_{1}$, z=+33.0) and upper speed quantile ($h_{2}$, z=+29.8) relative to the cell’s normal medians. The selected frame lies within the annotated anomalous interval. Although no object candidate is associated with the selected cell, the report characterizes the alarm region through its unusually high motion measurements.

**Figure 3:**
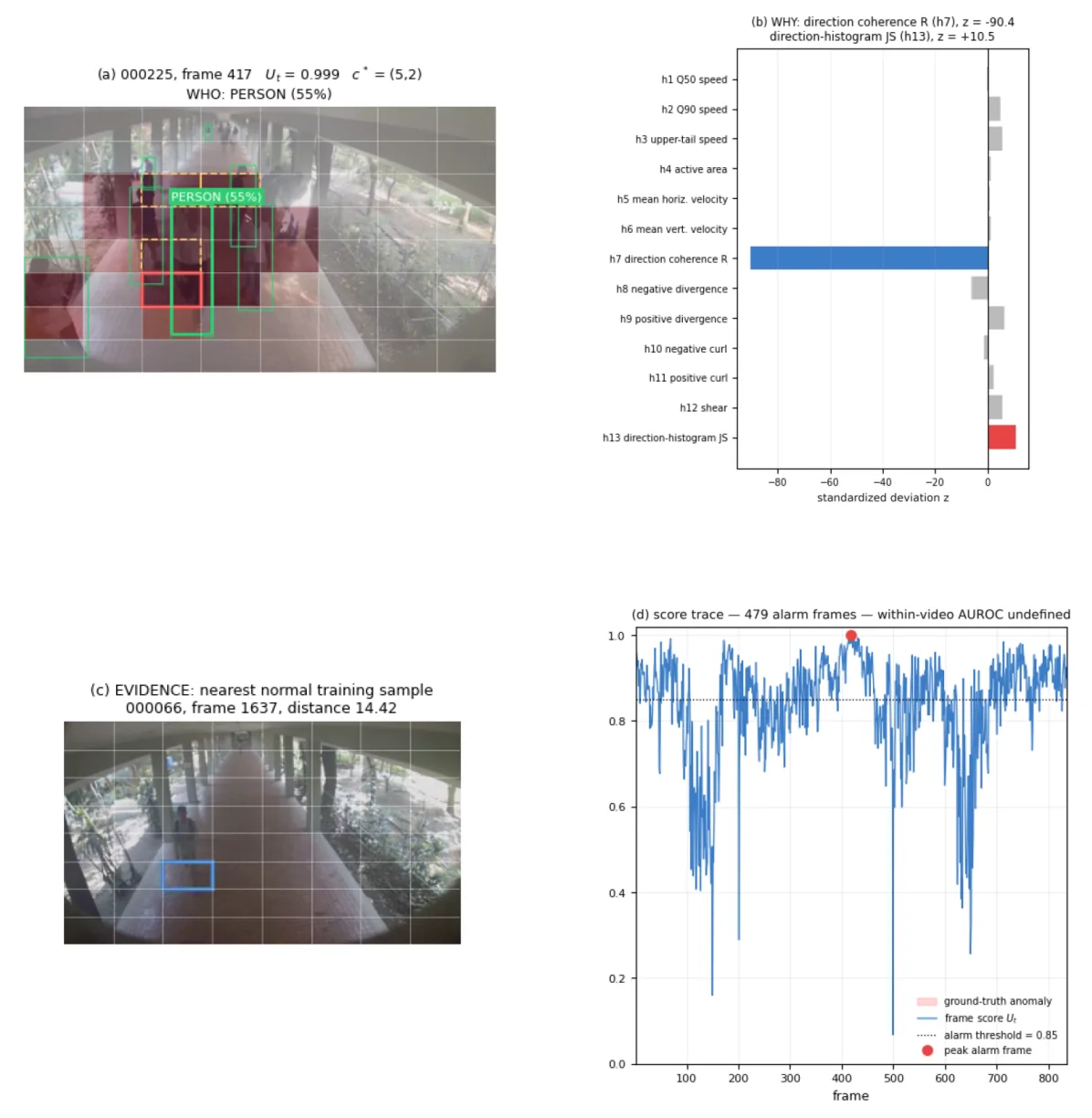
IITB-Corridor video 000225: complete explanation report at frame 417. Of the 835 normal-labeled frames, 479 reach the alarm threshold. Within-video AUROC is undefined for this single-class video, but its frames remain included in the pooled micro AUROC.

Figure 3 shows IITB-Corridor video 000225, whose 835 scored frames are all labeled normal. Nevertheless, 479 frames reach the alarm threshold, giving a within-video false-positive fraction of 57.4%. At frame 417, a person is associated with the selected region, and the largest deviations are reduced direction coherence ($h_{7}$, z=−90.4) and temporal change ($h_{13}$, z=+10.5). The nearest normal reference is training video 000066, frame 1637. This case shows that a kinematic departure and an associated person can occur during normal activity. The report presents the measurements behind the selected region, while the annotations identify the alarm as a false positive.

Appendix D presents reports for the highest- and lowest-performing Ped2 videos and the lowest-performing Avenue video. The score trace describes temporal discrimination. The selected region, descriptor profile, and normal reference provide an inspectable account of the reported motion.

## 6. Discussion

X-VAD addresses the research question by using explicit local motion descriptors for both anomaly scoring and kinematic reporting. Each report connects an alarm with its image region, measured deviations, and an observed normal reference. The method reaches 75.43% micro AUROC on IITB-Corridor, close to VideoPatchCore’s 76.4%. Its Ped2 and Avenue results are 85.78% and 84.42%, compared with the highest listed values of 99.3% and 93.7%. These gaps of 13.52 and 9.28 percentage points show that the evaluated motion-only configuration provides weaker frame-level discrimination on these two benchmarks.

The ablations help characterize this behavior. Removing deformation causes the largest group-level decrease on Ped2, while removing direction or temporal change has a greater effect on Avenue. Some descriptor removals improve AUROC, showing that the benefit of a measurement depends on the scene and the remaining representation. The fixed H13 configuration provides a common basis for evaluating these effects. Per-cell calibration and spatial aggregation also influence performance, while the tested changes in neighbor count and calibration-fold count have smaller effects.

The qualitative reports illustrate how the same representation describes detections and false alarms. Avenue video 03 shows elevated speed during an annotated anomalous interval. In IITB-Corridor video 000225, normal activity instead departs substantially from the local reference. Region-specific comparison, also used by EVAL [26], makes this departure interpretable in its spatial context. X-VAD expresses the comparison through explicit flow measurements and adds object information after scoring. An operator can therefore inspect the region, examine its descriptor profile, and compare it with a normal example from the same location.

This reporting function is distinct from maximizing detection accuracy. A stronger anomaly detector combined with an object detector may provide better discrimination and object context. Superiority over that combination has not been established for X-VAD. The present contribution is the shared kinematic representation linking the score and report. The reported descriptor ranking describes departures from normal medians, while scoring uses distances to neighboring normal vectors. The ranking is therefore descriptive rather than an additive attribution of the score. The observed AUROC gaps do not establish that interpretability causes a loss of accuracy.

### Limitations

Motion-only scoring has limited sensitivity to stationary objects and unusual appearance without displacement. Slow motion may fall below the active-motion threshold or resemble the normal reference. Post-alarm object annotation cannot recover an event that does not trigger the kinematic scorer. Perspective, occlusion, illumination changes, and camera shake can also affect optical flow. Spatial coverage is another constraint: only 24 Ped2 cells meet the memory requirements, and the four-cell mean requires four nonzero responses to reach the alarm threshold.

The evaluation covers three fixed-camera scenes, with descriptor and scoring ablations on all three scenes. Alternative flow estimators, controlled flow-error perturbations, and quantitative localization were not evaluated. Explanation faithfulness, sufficiency, and operator benefit also require dedicated evaluation. The descriptor ablations assess detection performance.

## 7. Conclusions

This study introduced X-VAD, a video anomaly detection method that produces kinematic reports. X-VAD compares 13 optical-flow descriptors with normal observations stored for each image cell. The same representation is used for anomaly scoring and for reports that show the alarm region, the descriptor deviations, and the nearest normal example. Post-alarm object annotation adds context without changing the score.

With a single configuration for all three benchmarks, X-VAD achieved micro AUROC values of 85.78% on Ped2, 84.42% on Avenue, and 75.43% on IITB-Corridor. The IITB-Corridor result is close to the 76.4% reported for VideoPatchCore. The Ped2 and Avenue results remain below the highest listed values of 99.3% and 93.7%, respectively. These published values were obtained under the original evaluation protocols of the respective studies. At the alarm threshold, the TPRs were 87.69%, 90.78%, and 62.73%, at FPRs of 41.46%, 46.32%, and 26.53%. With frame-by-frame processing on one GPU, the mean score-availability times were 50.0, 50.6, and 82.6 ms. Report generation, including YOLO-World inference, added 16.3–18.3 ms per alarm frame. The measured score-availability rate exceeded the frame rate only for Ped2, whose 10 fps is assumed. The ablations showed that descriptor contributions were scene-dependent. Among the scoring choices, spatial aggregation and calibration had the largest effects. Causal smoothing reduced the false-positive rate at the fixed alarm threshold in the tested settings. AUROC changed little when the assumed frame rate was varied within the evaluated ranges.

The main contribution is a shared kinematic representation of optical flow that serves both anomaly scoring and inspectable reporting, including the examination of false alarms. Future work will test the descriptors with other optical-flow estimators and under controlled flow perturbations.

## Figures and Tables

**Table 1:** Notation used in Section 3 and Section 4.

Symbol	Definition
$V_{\mathrm{tr}}$, $V_{\mathrm{te}}$	Normal training videos and test videos of a scene
$I_{t}$, $\left(I_{t},I_{t+1}\right)$	Frame *t* and the consecutive frame pair scored at index *t*
*W*, *H*, *D*, δt	Processed width and height (pixels), image diagonal, frame interval (s)
$F_{t}$, $f_{t}$, $m_{t}$	Raw flow (pixels), normalized flow (diagonal units per second), flow magnitude
*c*, $N_{C}$, $N_{P}$	Cell index, number of cells (64), pixels per cell
σ	Gaussian smoothing scale for spatial derivatives, σ=0.002D
$\tau_{c}$	Active-motion threshold of cell *c* (Section 4.2)
*A*, $A^{+}$	Active-pixel set of a cell and its dilated support
Δ	Temporal gap of the temporal descriptor, Δ=max{1,round(0.2fps)} frames
$h_{t,c,j}$, j=1,…,13	H13 descriptor *j* of cell *c* at pair *t*
$\mu_{c,j}$, ${\hat{s}}_{c,j}$	Training median and robust scale of descriptor *j* in cell *c*
$z_{t,c}\in R^{13}$	Standardized descriptor vector of cell *c*
$M_{c}$	Normal memory of cell *c* (standardized training vectors)
$d_{t,c}$, $p_{t,c}$	*k*-nearest-neighbor (*k*-NN) distance (k=3) and its empirical percentile
$T_{t}$	Set of the four cells with the largest $p_{t,c}$ at pair *t*
$U_{t}$, θ	Frame score (mean of the four largest $p_{t,c}$) and alarm threshold, θ=0.85
$c^{*}$	Highest-scoring cell of an alarm pair, $c^{*}=\arg\max_{c}p_{t,c}$
$E_{t}$, $J_{t}$	Explanation tuple of an alarm pair, indices of the two largest absolute standardized descriptor deviations
$r_{t}$, $o_{t}$	Nearest normal reference sample of $c^{*}$, associated object candidate (optional)

**Table 2:** H13 descriptors of an image cell.

Descriptor	Measurement	Definition	Interpretation
$h_{1}$	Median speed	$Q_{0.50}\left(m|A\right)$	Typical active-pixel speed
$h_{2}$	Upper speed quantile	$Q_{0.90}\left(m|A\right)$	Faster motion within the cell
$h_{3}$	Upper-tail speed	${\mathrm{TM}}^{+}\left(m|A\right)$	Extreme motion magnitude
$h_{4}$	Active fraction	$\left|A\right|/N_{P}$	Spatial extent of active motion
$h_{5}$	Horizontal motion	$\mathrm{mean}(f_{u}|A)$	Signed mean horizontal velocity
$h_{6}$	Vertical motion	$\mathrm{mean}(f_{v}|A)$	Signed mean vertical velocity
$h_{7}$	Direction coherence	$∥\sum_{A}{f∥}_{2}/\sum_{A}m$	Agreement among motion vectors
$h_{8}$	Lower divergence tail	${\mathrm{TM}}^{-}\left(\mathrm{div}|A^{+}\right)$	Lower tail of local expansion or contraction
$h_{9}$	Upper divergence tail	${\mathrm{TM}}^{+}\left(\mathrm{div}|A^{+}\right)$	Upper tail of local expansion or contraction
$h_{10}$	Lower curl tail	${\mathrm{TM}}^{-}\left(\mathrm{curl}|A^{+}\right)$	Lower-valued signed rotation
$h_{11}$	Upper curl tail	${\mathrm{TM}}^{+}\left(\mathrm{curl}|A^{+}\right)$	Higher-valued signed rotation
$h_{12}$	Upper shear tail	${\mathrm{TM}}^{+}\left(\mathrm{shear}|A^{+}\right)$	Strongest local shear
$h_{13}$	Temporal change	$\mathrm{JS}(P_{t},P_{t-\Delta })/\log2$	Change in the direction–activity distribution

*Q_q_*(·|*A*) is the *q*-quantile over active pixels, and TM^+^ and TM^−^ are upper and lower tail means. *N_p_* is the number of pixels in the cell. The normalized flow **f** = (*f_u_*, *f_v_*) and deformation fields are defined in Equations (2) and (4). *P_t_* is the direction–activity distribution in Equation (6), and JS is the Jensen–Shannon divergence in Equation (7). Speed is measured in image diagonals per second and deformation in s^−1^. Descriptors *h*_4_, *h*_7_, and *h*_13_ are dimensionless in [0, 1].

**Table 3:** Memory statistics of the three scenes after normal-only fitting.

Quantity	Ped2	Avenue	IITB-Corridor
Training pairs	2534	15,312	301,791
Motion-valid cell observations (%)	23.96	27.07	6.83
Scorable cells (of 64)	24	64	64
Calibration folds	4	4	4

**Table 4:** Dataset configuration after preprocessing. The lower rows give constants derived from image geometry and frame rate.

Quantity	Ped2	Avenue	IITB-Corridor
Training/test videos	16/12	16/21	208/150
Training/test frames	2550/2010	15,328/15,324	301,999/181,567
Native image size	360×240	640×360	1920×1080
Processed image size	360×240	640×360	1024×576
Frame rate used (fps)	10	25	30
Cell size (pixels)	45×30	80×45	128×72
Image diagonal *D* (pixels)	432.7	734.3	1174.9
Smoothing scale σ (pixels)	0.87	1.47	2.35
Dilation radius ⌈σ⌉ (pixels)	1	2	3
Temporal gap Δ (frames)	2	5	6
Threshold lower bound 0.3/(Dδt) (s^−1^)	0.00693	0.01021	0.00766

**Table 5:** Online runtime of X-VAD with a batch size of one on a single NVIDIA A100-SXM4-40GB GPU. Timings are reported as mean (95th percentile) over 1000 test pairs per dataset. Report times cover alarm frames only; combined times are summed per alarm frame.

Quantity	Ped2	Avenue	IITB-Corridor
Processed image size (pixels)	360×240	640×360	1024×576
Frame reading (ms)	0.7 (0.7)	0.5 (0.7)	17.7 (18.8)
Optical flow, SEA-RAFT (ms)	40.5 (41.7)	40.7 (42.3)	69.2 (69.6)
H13 extraction (ms)	8.6 (8.8)	8.8 (9.0)	11.4 (12.1)
Memory lookup and scoring (ms)	0.93 (0.97)	1.06 (1.11)	1.99 (2.03)
Score-availability time (ms)	50.0 (51.4)	50.6 (52.5)	82.6 (83.7)
Score-availability rate (frames/s)	20.0	19.8	12.1
Alarm frames among timed pairs	845	436	166
Report generation per alarm frame (ms)	16.4 (17.0)	16.3 (16.9)	18.3 (19.5)
YOLO-World inference and object association (ms)	15.5 (16.1)	15.4 (15.9)	17.3 (18.4)
Score availability and report, alarm frames (ms)	66.4 (68.3)	67.0 (69.5)	100.5 (102.2)
Peak GPU memory allocated (GB)	0.95	1.75	1.89

**Table 6:** Frame-level AUROC (%) and alarm rates at θ=0.85 (%) on the three benchmarks. The macro average is taken over the videos that contain both normal and anomalous frames (8 of 12, 19 of 21, and 134 of 150 test videos). Micro AUROC, TPR, and FPR pool all annotated test videos.

Metric	Ped2	Avenue	IITB-Corridor
Micro AUROC, all annotated test videos	85.78	84.42	75.43
Macro AUROC, videos with both labels	89.22	86.29	74.34
TPR at θ=0.85, all annotated test videos	87.69	90.78	62.73
FPR at θ=0.85, all annotated test videos	41.46	46.32	26.53

**Table 7:** Published frame-level AUROC (%) and X-VAD results.

Method	Year	Input	Postprocessing	Ped2	Avenue	IITB-Corridor
Future-frame prediction [16]	2018	R	V	95.4	85.1	N/A
MemAE [17]	2019	R	V	94.1	83.3	N/A
MNAD, prediction [18]	2020	R	V	97.0	88.5	N/A
MTP [10]	2020	P	NS	N/A	82.85	67.12
EVAL [26]	2023	R	NS	N/A	86.02	N/A
ViT-STRA [21]	2024	R	NS	95.6	86.8	N/A
VideoPatchCore [24]	2024	R+O+D	V ^†^, S	N/A	92.8	76.4
HGNF [23]	2024	P	NS	N/A	N/A	72.4
AI-VAD [42]	2025	O+F+P+D	S	99.1	93.7	N/A
Unified GAN, flow + RGB [19]	2025	R+F	NS	96.0	N/A	N/A
MS-UACL [20]	2026	R	V ^†^	97.3	89.6	N/A
SETJiP, CTJiP [22]	2026	R+O	V, S	99.3	93.4	N/A
X-VAD	—	F	None	85.78	84.42	75.43

Input of the anomaly score: R, RGB or grayscale frames; F, optical flow; P, human pose; O, object detections; D, pretrained deep features. Postprocessing stated in the original publication: V, per-video score normalization; S, temporal smoothing; NS, neither stated. ^†^ Normalization is stated, but its scope is not specified. N/A, not reported. Section 5.7 evaluates X-VAD with causal smoothing.

**Table 8:** Ablation study: frame-level micro AUROC (%) and change from the full model in percentage points, computed before rounding. One component is changed at a time, with all other constants as in Section 4 and Appendix A.

	Ped2		Avenue		IITB-Corridor	
Setting	AUROC	Change	AUROC	Change	AUROC	Change
Full H13 (k=3, top-4, 4 folds)	85.78	—	84.42	—	75.43	—
*Descriptor group removed*						
Speed and extent ($h_{1}$–$h_{4}$)	84.52	−1.26	84.44	+0.02	75.37	−0.06
Direction ($h_{5}$–$h_{7}$)	90.12	+4.33	81.80	−2.62	73.81	−1.63
Deformation ($h_{8}$–$h_{12}$)	83.46	−2.32	85.72	+1.30	75.62	+0.19
Temporal change ($h_{13}$)	87.01	+1.22	81.93	−2.49	75.62	+0.19
*Number of aggregated cells*						
Top-1	71.18	−14.60	84.13	−0.29	74.00	−1.43
Top-2	81.15	−4.63	85.02	+0.60	74.95	−0.48
Top-8	84.67	−1.11	81.55	−2.87	75.16	−0.28
*Number of nearest neighbors*						
k=1	86.22	+0.44	84.11	−0.32	75.55	+0.12
k=5	85.39	−0.39	84.55	+0.13	75.34	−0.10
*Calibration*						
2 folds	85.80	+0.02	84.55	+0.13	75.58	+0.14
8 folds	85.81	+0.03	84.36	−0.06	75.37	−0.07
Leave-self-out	86.13	+0.35	84.39	−0.03	75.24	−0.19
None (raw *k*-NN distance)	70.48	−15.30	83.30	−1.13	74.52	−0.91

**Table 9:** Individual-descriptor ablation: frame-level micro AUROC (%) and change from the full model in percentage points, computed before rounding. Each row removes one H13 coordinate and keeps the full model’s standardization statistics and validity rules.

		Ped2		Avenue		IITB-Corridor	
Removed	Descriptor	AUROC	Change	AUROC	Change	AUROC	Change
$h_{1}$	Median speed	85.59	−0.19	84.65	+0.23	75.47	+0.04
$h_{2}$	Upper speed quantile	85.63	−0.15	84.49	+0.07	75.47	+0.03
$h_{3}$	Upper-tail speed	85.76	−0.02	84.50	+0.08	75.46	+0.03
$h_{4}$	Active fraction	85.34	−0.44	84.03	−0.39	75.27	−0.16
$h_{5}$	Horizontal motion	84.60	−1.18	84.48	+0.06	75.07	−0.36
$h_{6}$	Vertical motion	86.16	+0.38	82.84	−1.59	75.15	−0.29
$h_{7}$	Direction coherence	89.20	+3.42	83.40	−1.02	74.86	−0.57
$h_{8}$	Lower divergence tail	85.60	−0.19	84.64	+0.22	75.47	+0.04
$h_{9}$	Upper divergence tail	85.50	−0.28	84.66	+0.24	75.47	+0.04
$h_{10}$	Lower curl tail	85.28	−0.50	84.63	+0.21	75.48	+0.05
$h_{11}$	Upper curl tail	85.32	−0.46	84.68	+0.26	75.46	+0.03
$h_{12}$	Upper shear tail	85.57	−0.21	84.53	+0.10	75.46	+0.02
$h_{13}$	Temporal change	87.01	+1.22	81.93	−2.49	75.62	+0.19

**Table 10:** Frame-level micro AUROC (%) and false-positive rate at the alarm threshold θ=0.85 (FPR, %) with a causal moving average of the frame scores.

Dataset	None	≈0.3 s	≈0.5 s	≈1.1 s
*Micro AUROC*				
Ped2	85.78	87.46	87.77	87.66
Avenue	84.42	84.67	84.23	82.83
IITB-Corridor	75.43	76.30	76.39	76.27
*FPR at θ=0.85*				
Ped2	41.46	39.50	36.13	32.21
Avenue	46.32	44.69	44.56	44.70
IITB-Corridor	26.53	24.01	23.49	22.72

Window lengths correspond to 3, 5, and 11 frames (Ped2), 8, 13, and 28 frames (Avenue), and 9, 15, and 33 frames (IITB-Corridor). The main results in Table 6 and Table 7 use no smoothing. Durations follow the frame rates in Table 4.

**Figure A1:**
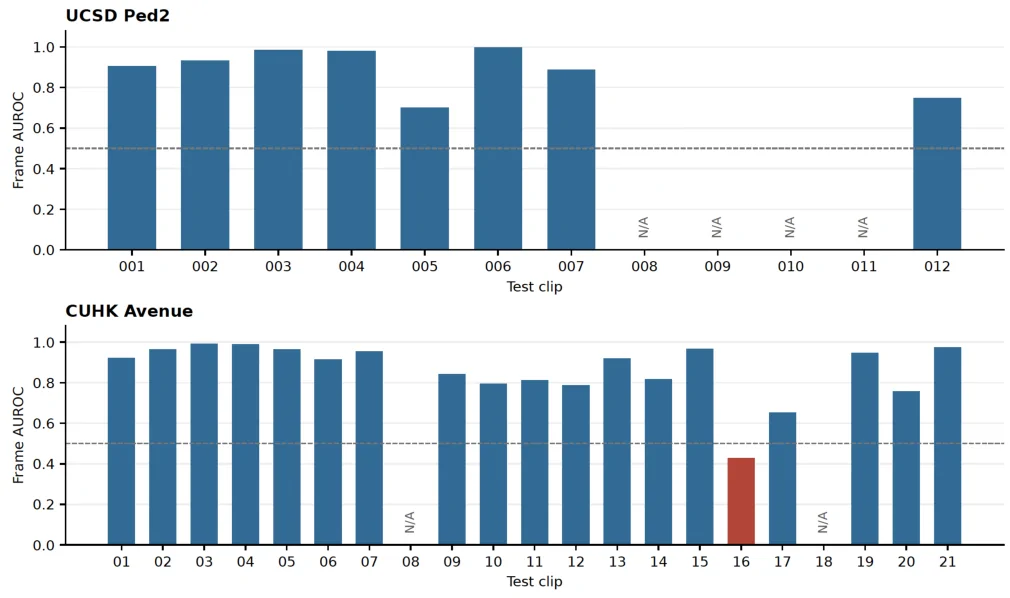
Within-video AUROC on Ped2 and Avenue. N/A denotes videos whose frames all carry the same ground-truth label.

**Figure A2:**
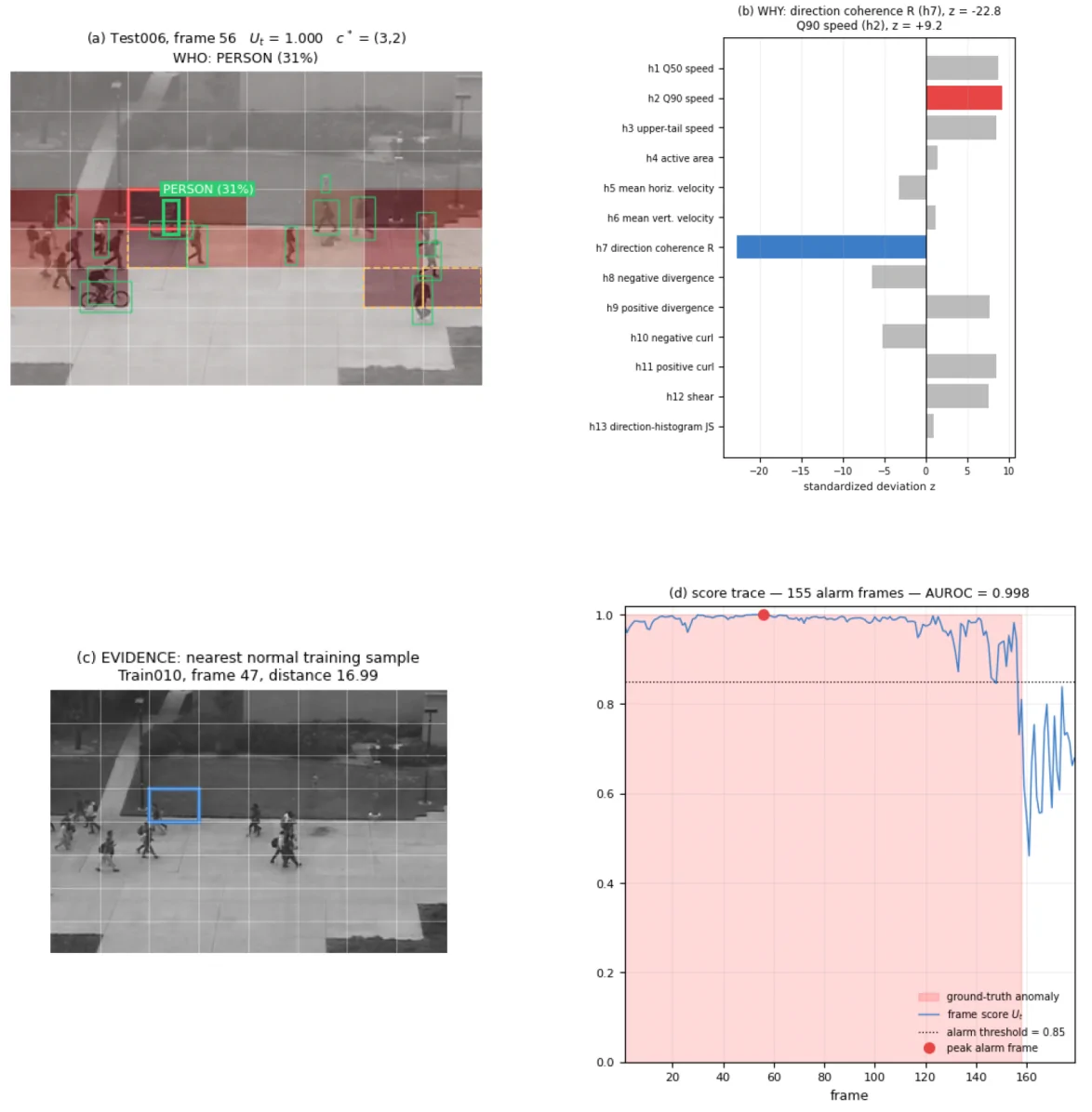
Ped2 Test006: highest within-video AUROC (99.8%), with the report at frame 56.

**Figure A3:**
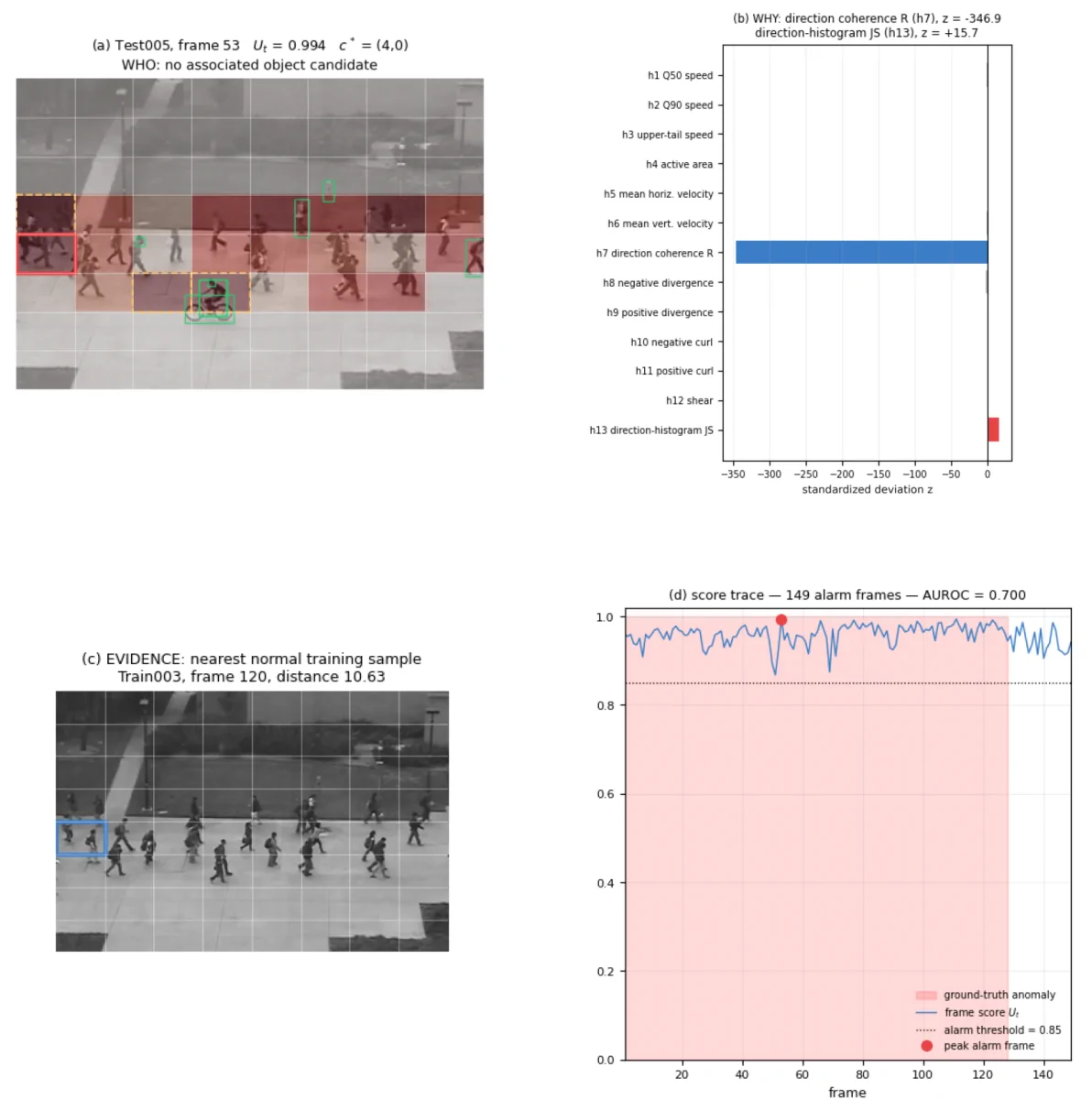
Ped2 Test005: lowest within-video AUROC (70.0%), with persistent alarms and no associated object candidate at frame 53.

**Figure A4:**
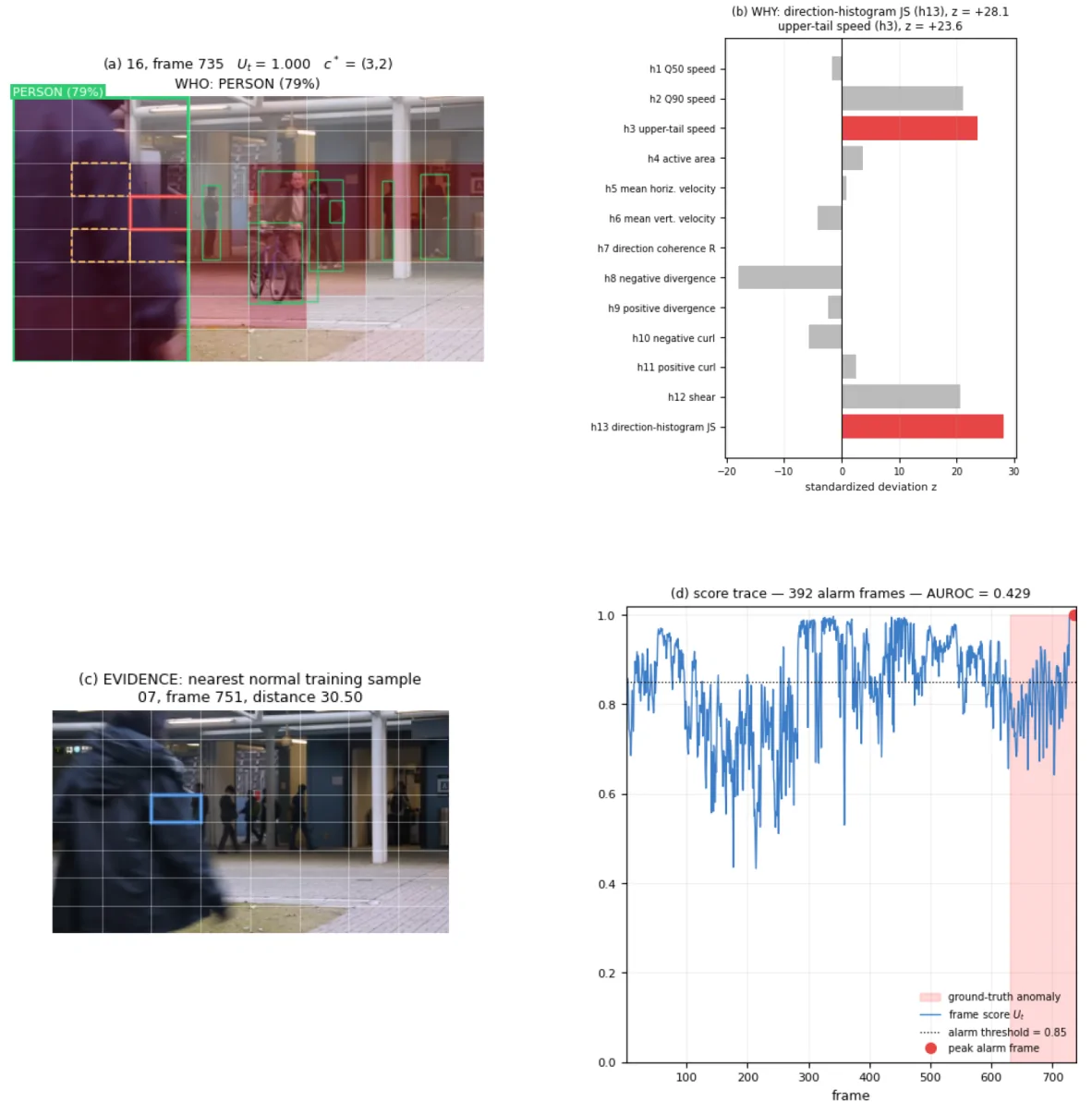
Avenue video 16: lowest within-video AUROC (42.9%), with the report at frame 735.

## Data Availability

Publicly available datasets were analyzed in this study. These data can be found here: UCSD Ped2, http://www.svcl.ucsd.edu/projects/anomaly/dataset.htm (accessed on 17 September 2026); CUHK Avenue, https://www.cse.cuhk.edu.hk/leojia/projects/detectabnormal/dataset.html (accessed on 17 September 2026); and IITB-Corridor, https://rodrigues-royston.github.io/Multi-timescale_Trajectory_Prediction/ (accessed on 17 September 2026). The source code of X-VAD is available from the corresponding author upon reasonable request, because it is part of an ongoing doctoral dissertation.

## Appendix A. Implementation Details

This appendix specifies the implementation of X-VAD evaluated in the main text. Each training and test video is processed independently. The first retained frame has no score, and every following frame receives the score of the pair ending at that frame.

### Appendix A.1. Input Processing

The frame-rate parameter was set to 10 fps for Ped2 and 30 fps for IITB-Corridor, whose videos are reported at 25 fps. For Avenue, the decoded frame rate of 25 fps was used. IITB-Corridor images are resized by area interpolation from 1920×1080 to 1024×576. The first frame is dropped if its mean intensity is below 8. When the annotation array then exceeds the retained frame count by one, its first entry is also dropped. Otherwise, the annotation length is required to equal the number of retained frames. Positive annotation values are binarized to one. Avenue frame labels indicate whether any pixel in the released annotation mask is positive. Ped2 uses the released frame intervals, checked against the mask-derived frame labels.

### Appendix A.2. Feature Extraction and Calibration

SEA-RAFT uses the spring-M configuration and checkpoint MemorySlices/Tartan-C-T-TSKH-spring540x960-M. Four refinement iterations are applied with scale zero, corresponding to the processed resolution. Gaussian smoothing uses reflection padding and radius ⌈3σ⌉, where σ=0.002D. Derivatives use central differences in the image interior and one-sided differences at boundaries. Mask dilation is applied before partitioning the image into cells, allowing the support to cross cell boundaries.

Magnitude calibration samples 16 pixels per cell in each selected training pair, using fixed indices and cyclic batch-dependent offsets. Within each training video, pairs are sampled at a stride of $\max\{1,\left\lfloor N_{\mathrm{pairs}}/5000\right\rfloor \}$. Here, $N_{\mathrm{pairs}}$ is the total number of pairs across all training videos of the scene. Magnitude calibration uses 2534 pairs on Ped2, 5111 on Avenue, and 5128 on IITB-Corridor. The IITB-Corridor pair stride is 60.

Quantiles use linear interpolation. Tail means are zero for supports smaller than eight pixels. Descriptors $h_{1}$–$h_{7}$ use motion validity, $h_{8}$–$h_{12}$ use dilated-mask validity, and $h_{13}$ becomes valid after the temporal delay, independently of the first two flags. The raw active fraction can be nonzero for fewer than eight active pixels, but its standardized coordinate is then zero. Direction histograms use linear assignment to adjacent circular bins weighted by active flow magnitude. A constant of $10^{-12}$ is added to their normalization denominator and to the direction-coherence denominator. The mixture denominator used in the Jensen–Shannon divergence is floored at $10^{-12}$. Zero-mass terms contribute zero, natural logarithms are used, and no square root is taken.

For each cell and descriptor, at least ten valid training values are required to estimate the median and the scaled MAD. Otherwise, both are set to zero. The scale floor is the 10th percentile of positive scaled MADs across the scene’s cells and descriptors, or $10^{-6}$ if none is positive. The final scale is the maximum of the scaled MAD and this floor.

### Appendix A.3. Memory and Score Computation

Each cell requires at least 100 motion-valid training vectors. Memories retain at most 40,000 vectors through evenly spaced integer indices. Four folds are assigned by sorted training-video index modulo four. Held-out motion-valid vectors are compared with vectors in the remaining folds, using the same memory cap. A fold contributes calibration distances only if at least 100 memory vectors are available for the cell. A cell is scorable when a full memory and at least one calibration distance are available.

Distances are computed by exhaustive Euclidean search, and the three smallest distances are averaged. The percentile uses strict inequality for ties (equivalent to a left insertion index) and the add-one correction given in Equation (10). Thresholds and scaling statistics are fitted once on the entire training split. Fold separation applies only to neighbor membership. Test queries use the full memory. The calibration therefore supplies an empirical distance reference rather than a conformal probability or a prescribed false-alarm rate (Section 4.3). Nonscorable or motion-invalid cells contribute zero, and the denominator of the frame-score average remains four.

The descriptor memories contain at most 64×40,000 vectors of 13 single-precision values, requiring up to 133.1 MB for the vectors alone. This bound excludes model weights, intermediate tensors, calibration arrays, and search buffers. Building the IITB-Corridor memories and calibration distributions from extracted descriptors takes 13 s.

### Appendix A.4. Runtime Measurement and Score Consistency

The timed pairs were drawn from Ped2 Test001–Test007, Avenue video 01, and IITB-Corridor videos 000209–000211. Over the 1000 timed pairs per dataset, the maximum absolute differences between online and stored offline scores are approximately 0.032, 0.059, and 0.067. The alarm decisions at θ=0.85 agree for 99.7%, 99.9%, and 99.7% of these pairs. For the same pairs, passing the stored descriptors to the online scorer gives maximum absolute differences of approximately $5.0\times 10^{-4}$, $8.4\times 10^{-5}$, and $3.4\times 10^{-5}$ from the stored scores, with identical alarm decisions.

The main experiments were run on one NVIDIA A100-SXM4-80GB graphics processing unit (GPU). With batches of eight pairs, SEA-RAFT inference takes 6.3, 14.0, and 36.1 ms per pair at the Ped2, Avenue, and IITB-Corridor resolutions, respectively. These are batch-averaged inference times. The extraction pipeline, including optical flow, H13, and validity flags, processes approximately 59 pairs/s on Avenue and 17.5 pairs/s on IITB-Corridor. These rates describe offline throughput and exclude memory lookup and post-alarm annotation and reporting.

### Appendix A.5. Software

The main experiments used an NVIDIA A100-SXM4-80GB GPU, Python 3.13.15, PyTorch 2.11.0 (CUDA 12.8), OpenCV 5.0.0, NumPy 2.1.3, Ultralytics 8.4.142, scikit-learn 1.6.1, and SciPy 1.16.3. Sampling and fold assignment use fixed indices. Benchmarking in cuDNN is disabled, its deterministic mode is enabled, and the PyTorch seed is set to zero. The deterministic-algorithms check is enabled in warning mode, so that an operation without a deterministic kernel raises a warning rather than an error [43]. The YOLO-World checkpoint is yolov8m-worldv2.pt. The additional ablation and frame-rate experiments used PyTorch 2.11.0 with CUDA 13.0 on an NVIDIA A100-SXM4-80GB GPU. The new Ped2 flow extraction for the frame-rate runs used an NVIDIA Tesla T4 GPU instead. Their recorded NumPy, scikit-learn, and SciPy versions were unchanged, and the frame-rate runs recorded OpenCV 4.14.0. The online runtime measurements in Table 5 used an NVIDIA A100-SXM4-40GB GPU, Python 3.13.15, PyTorch 2.11.0 (CUDA 13.0), OpenCV 4.14.0, NumPy 2.1.3, Ultralytics 8.4.142, scikit-learn 1.6.1, and SciPy 1.16.3. They used the same SEA-RAFT checkpoint, YOLO-World weights, and determinism settings as the main experiments.

### Appendix A.6. Pseudocode of the Subroutines

The following subroutines specify input processing, descriptor computation, and normal-only fitting for Algorithm 1. Algorithm A1 reads and aligns one video. Algorithm A2 computes H13 and the validity flags for one frame pair. Algorithm A3 fits the normal reference for one scene. All constants are defined in Section 4 and Appendix A.

**Algorithm A1** Input processing and label alignment for one video	
**Require:** Frame sequence $I_{1},\ldots ,I_{T_{0}}$ at native size; processed size (W,H); test flag; frame labels $\ell_{1},\ldots ,\ell_{L}$ (test videos only)	
**Ensure:** Processed frames $I_{1},\ldots ,I_{T}$; frame pairs $\left(I_{t},I_{t+1}\right)$, t=1,…,T−1; aligned labels $y_{2},\ldots ,y_{T}$ (test videos only)	
1: **if** the mean intensity of $I_{1}$ is below 8 (IITB-Corridor) **then**	
2: drop $I_{1}$	▹ leading black frame
3: **end if**	
4: **for all** remaining frames **do**	
5: **if** a processed size smaller than the native size is configured **then**	
6: resize to (W,H) by area interpolation	▹ IITB-Corridor: 1920×1080→1024×576
7: **else if** the native size is not a multiple of 8 **then**	
8: crop the bottom and right borders to the largest multiple of 8	▹ not triggered for the three datasets
9: **end if**	
10: convert to RGB	
11: **end for**	
12: form the T−1 consecutive pairs within the video; pairs do not cross video boundaries	
13: **if** test video **then**	
14: binarize the labels: $\ell_{i}\leftarrow 1\left[\ell_{i}>0\right]$	▹ Ped2: released frame intervals; Avenue: any positive pixel in the released mask; IITB-Corridor: released .npy array
15: **if** $I_{1}$ was dropped and L=T+1 **then**	
16: drop $\ell_{1}$; L←L−1	
17: **end if**	
18: require L=T	
19: $y_{t+1}\leftarrow \ell_{t+1}$ for t=1,…,T−1▹ score of pair *t* is attributed to frame t+1; frame 1 receives no score	
20: **end if**	

**Algorithm A2** H13 descriptors and validity flags for one frame pair	
**Require:** Raw flow $F_{t}$ (pixels); geometry *D*, δt, σ=0.002D; thresholds $\tau_{c}$; distribution $P_{t-\Delta }$ of the same video (if t>Δ)	
**Ensure:** $h_{t,c,1..13}$, flags ${\left(\mathrm{motion}\_\mathrm{valid},\mathrm{dcs}\_\mathrm{valid},\mathrm{js}\_\mathrm{valid}\right)}_{t,c}$ (validity of the speed/direction, divergence–curl–shear, and temporal descriptors), and $P_{t}$ for all cells *c*	
1: $f\leftarrow F_{t}/\left(D\;\delta t\right)$; $m\leftarrow {∥f∥}_{2}$	▹ Equation (2)
2: $\bar{f}\leftarrow G_{\sigma }*f$ with reflection padding and kernel radius ⌈3σ⌉	
3: $J_{11},J_{12},J_{21},J_{22}\leftarrow D\cdot$ partial derivatives of $\bar{f}$ (central; one-sided at the border)	▹ Equation (3)
4: $\mathrm{div}\leftarrow J_{11}+J_{22}$; $\mathrm{curl}\leftarrow J_{21}-J_{12}$; $\mathrm{shear}\leftarrow \sqrt{{\left(J_{11}-J_{22}\right)}^{2}+{\left(J_{12}+J_{21}\right)}^{2}}$	▹ Equation (4)
5: $A\leftarrow \{m>\tau_{c(\cdot )}\}$ over the whole image; $A^{+}\leftarrow$ square maximum filter of *A* with half-width ⌈σ⌉	
6: **for all** cells *c* **do**	
7: $A_{c}\leftarrow A\cap c$; $A_{c}^{+}\leftarrow A^{+}\cap c$; $N_{A}\leftarrow \left|A_{c}\right|$; $N_{A^{+}}\leftarrow \left|A_{c}^{+}\right|$	
8: $\mathrm{motion}\_\mathrm{valid}\leftarrow N_{A}\geq 8$; $\mathrm{dcs}\_\mathrm{valid}\leftarrow N_{A^{+}}\geq 8$	
9: $h_{1}\leftarrow Q_{0.50}\left(m|A_{c}\right)$; $h_{2}\leftarrow Q_{0.90}\left(m|A_{c}\right)$ (linear interpolation); $h_{3}\leftarrow {\mathrm{TM}}^{+}\left(m|A_{c}\right)$; $h_{4}\leftarrow N_{A}/N_{P}$	
10: $h_{5}\leftarrow \mathrm{mean}\left(f_{u}|A_{c}\right)$; $h_{6}\leftarrow \mathrm{mean}\left(f_{v}|A_{c}\right)$; $h_{7}\leftarrow {∥\sum_{A_{c}}f∥}_{2}/\left(\sum_{A_{c}}m+10^{-12}\right)$	
11: **if not** motion_valid **then**	
12: $h_{1},h_{2},h_{5},h_{6},h_{7}\leftarrow 0$	▹$h_{3}$ is zero by the tail-mean rule; the raw $h_{4}$ is retained
13: **end if**	
14: $h_{8},h_{9}\leftarrow {\mathrm{TM}}^{-},{\mathrm{TM}}^{+}\left(\mathrm{div}|A_{c}^{+}\right)$; $h_{10},h_{11}\leftarrow {\mathrm{TM}}^{-},{\mathrm{TM}}^{+}\left(\mathrm{curl}|A_{c}^{+}\right)$; $h_{12}\leftarrow {\mathrm{TM}}^{+}\left(\mathrm{shear}|A_{c}^{+}\right)$	
where ${\mathrm{TM}}^{\pm }\left(x|S\right)$ averages the min{max(⌈0.01|S|⌉,8),|S|} largest (smallest) values and is 0 if |S|<8	
15: H← eight-bin circular histogram of $\mathrm{atan2}(f_{v},f_{u})$ over $A_{c}$, weight *m*, linear split between adjacent bins, normalized by $\left(\sum H+10^{-12}\right)$	
16: $P_{t}\leftarrow \left[\;1-h_{4},\;h_{4}H_{1},\ldots ,h_{4}H_{8}\;\right]$	▹ Equation (6)
17: **if** t>Δ **then**	
18: $h_{13}\leftarrow \mathrm{JS}\left(P_{t},P_{t-\Delta }\right)/\log2$ with $M=\frac{1}{2}\left(P_{t}+P_{t-\Delta }\right)$	▹ Equation (7)
terms $p\log(p/\max\left(M,10^{-12}\right))$ for p>0, zero otherwise; js_valid← true	
19: **else**	
20: $h_{13}\leftarrow 0$; js_valid← false	
21: **end if**	
22: **end for**	

**Algorithm A3** Normal-only fitting for one scene: thresholds, scaling statistics, memories, and calibration distributions	
**Require:** Normal training videos $V_{1},\ldots ,V_{n}$ with $N_{\mathrm{pairs}}$ pairs in total; constants k=3, MIN_MEM=100, MAX_MEM=40,000, four folds	
**Ensure:** $\tau_{c}$; $\mu_{c,j}$, ${\hat{s}}_{c,j}$; memories $M_{c}$; sorted reference distances $\left\{d_{i}^{\left(c\right)}\right\}$; scorable set	
*Active-motion thresholds*	
1: $\mathrm{SUB}\leftarrow \max\{1,\left\lfloor N_{\mathrm{pairs}}/5000\right\rfloor \}$; stride $\leftarrow \lfloor N_{P}/16\rfloor$	
2: **for all** videos $V_{i}$ and pairs *t* with (t−1)modSUB=0, processed in batches b=0,1,… **do**	
3: $m\leftarrow ∥F_{t}{/\left(D\;\delta t\right)∥}_{2}$; for every cell take the 16 pixels at positions {p·stride+(bmodstride):p=0,…,15}	
4: **end for**	
5: **for all** cells *c* **do**	
6: $\tau_{c}\leftarrow \max\left\{\mathrm{median}\left({\tilde{m}}_{c}\right)+3\;\left(1.4826\right)\mathrm{MAD}\left({\tilde{m}}_{c}\right),\;0.3/\left(D\;\delta t\right)\right\}$	▹ Equation (5)
7: **end for**	
*Descriptors and robust scaling*	
8: run Algorithm A2 on all training pairs; collect $h_{t,c,j}$ and the validity flags	
9: **for all** cells *c* and descriptors *j* **do**	
10: x← values of $h_{\cdot ,c,j}$ whose group flag is valid (j≤7: motion_valid; 8≤j≤12: dcs_valid; j=13: js_valid)	
11: **if** |x|≥10 **then**	
12: $\mu_{c,j}\leftarrow \mathrm{median}\left(x\right)$; $s_{c,j}\leftarrow 1.4826\mathrm{MAD}\left(x\right)$	
13: **else**	
14: $\mu_{c,j},s_{c,j}\leftarrow 0$	
15: **end if**	
16: **end for**	
17: ε← 10th percentile of $\left\{s_{c,j}|s_{c,j}>0\right\}$ ($10^{-6}$ if none); ${\hat{s}}_{c,j}\leftarrow \max\left\{s_{c,j},\varepsilon \right\}$	
18: $z_{t,c,j}\leftarrow \left(h_{t,c,j}-\mu_{c,j}\right)/{\hat{s}}_{c,j}$ where the group flag is valid, else 0	▹ Equation (8)
*Out-of-fold calibration and memories*	
19: assign video $V_{i}$ to fold (i−1)modmin{4,n}	
20: **for all** folds *f* and cells *c* **do**	
21: R← motion-valid vectors of *c* not in fold *f*, evenly spaced subsample if |R|>MAX_MEM; Q← motion-valid vectors of *c* in fold *f*	
22: **if** |R|≥MIN_MEM and Q≠∅ **then**	
23: $d_{q}\leftarrow$ mean distance of $z_{q}$ to its *k* nearest vectors in R for all q∈Q	▹ Equation (9), exact search
24: **end if**	
25: **end for**	
26: **for all** cells *c* **do**	
27: $\{d_{i}^{\left(c\right)}\}\leftarrow$ sorted out-of-fold distances of *c*	
28: $M_{c}\leftarrow$ all motion-valid vectors of *c*, evenly spaced subsample if more than MAX_MEM, provided their number is at least MIN_MEM	
29: *c* is scorable if and only if $M_{c}$ exists and $\{d_{i}^{\left(c\right)}\}\neq \emptyset$	
30: **end for**	

## Appendix B. Additional Evaluation Results

Table A1 reports the sensitivity of the frame-level micro AUROC to the assumed frame rate, which is discussed in Section 5.7.

The reference micro AUROCs of the frame-rate runs are 85.93% for Ped2, 84.42% for Avenue, and 75.43% for IITB-Corridor. Ped2 and Avenue use a new flow extraction shared across rates, so their frame scores are not identical to those of the main experiment. IITB-Corridor uses stored descriptors and half-precision direction–activity distributions. Reconstructing $h_{13}$ changes its 30 fps reference AUROC by less than 0.001 percentage points. This reconstructed reference also supplies the IITB-Corridor smoothing results.

Table A2 reports macro-averaged AUROC, computed as the unweighted mean of the within-video AUROCs over videos containing both normal and anomalous frames. Within-video AUROC is undefined for single-class videos.

**Table A1:** Change in micro AUROC (percentage points) for different assumed frame rates with unchanged frame pairs. Changes are relative to the reference rate (*) of each run and computed before rounding.

Dataset	Assumed Rate (fps)	Change
Ped2	5	−0.93
	8	−0.28
	10 *	—
	15	+0.72
	20	+0.81
Avenue	12.5	+0.06
	20	+0.25
	25 *	—
	30	−0.07
	37.5	−0.37
	50	−0.44
IITB-Corridor	15	−0.12
	24	−0.04
	25	−0.03
	30 *	—
	45	+0.03
	60	+0.12

**Table A2:** Macro-averaged AUROC (%).

Metric	Ped2	Avenue	IITB-Corridor
Macro AUROC	89.22	86.29	74.34
Videos in the average (of all test videos)	8 of 12	19 of 21	134 of 150

Figure A1 summarizes the variation across Ped2 and Avenue videos. Table A3 and Table A4 give their per-video results.

**Table A3:** UCSD Ped2: within-video AUROC (%). N/A denotes videos whose frames all carry the same ground-truth label.

Video	Scored Frames	Anomaly Fraction	AUROC (%)
Test001	179	0.6704	90.58
Test002	179	0.4804	93.32
Test003	149	0.9732	98.62
Test004	179	0.8380	98.00
Test005	149	0.8591	70.01
Test006	179	0.8827	99.79
Test007	179	0.7542	88.67
Test008	179	1.0000	N/A
Test009	119	1.0000	N/A
Test010	149	1.0000	N/A
Test011	179	1.0000	N/A
Test012	179	0.5196	74.73

**Table A4:** CUHK Avenue: within-video AUROC (%). N/A denotes videos whose frames all carry the same ground-truth label.

Video	Scored Frames	Anomaly Fraction	AUROC (%)
01	1438	0.2837	92.11
02	1210	0.0736	96.48
03	922	0.0944	99.06
04	946	0.0983	98.87
05	1006	0.3161	96.52
06	1282	0.3346	91.36
07	604	0.2036	95.33
08	35	1.0000	N/A
09	1174	0.2462	84.16
10	840	0.1583	79.55
11	471	0.3057	81.16
12	1270	0.1496	78.63
13	548	0.1369	92.02
14	506	0.1462	81.80
15	1000	0.0900	96.64
16	739	0.1475	42.92
17	425	0.9012	65.37
18	293	1.0000	N/A
19	247	0.5668	94.60
20	272	0.5662	75.66
21	75	0.7067	97.34

## Appendix C. Semantic Vocabulary

The 31 prompts are as follows: person, person lying on the ground, person wearing a face mask, person wearing a balaclava, person wearing a helmet, group of people, bicycle, motorcycle, scooter, skateboard, car, bus, truck, gun, pistol, rifle, knife, machete, axe, hammer, baseball bat, crowbar, scissors, backpack, handbag, suitcase, abandoned bag, cardboard box, fire, smoke, and dog. The prompts define the detector vocabulary. They do not imply that each category occurs in the test videos. A category such as “abandoned bag” is a detector prediction. Abandonment itself requires temporal evidence.

## Appendix D. Complete Explanation Examples

Report figures and their descriptions use zero-based frame and grid row–column indices. The mathematical notation uses one-based indices. The descriptor plots show the standardized deviations *z* defined in Equation (8). For divergence and curl, “negative” and “positive” label the lower and upper tails defined in Table 2. The raw tail values are not restricted to those signs.

The figures below show complete explanation reports. Panels provide the alarm frame and selected regions, signed descriptor deviations, the nearest normal reference, and the unsmoothed frame-score trace. The panel labels WHO, WHY, and EVIDENCE denote the associated object candidate, largest marginal descriptor deviations, and nearest normal training sample, respectively. WHY describes deviations from normal medians and does not indicate causal attribution. Normal references use one nearest neighbor for display, whereas the detection score uses three (Section 4.4). Grid regions are overlaid on the second frame without flow warping and should be interpreted as approximate spatial associations.

The Ped2 examples cover the highest and lowest within-video AUROC: Test006 (99.8%, Figure A2) and Test005 (70.0%, Figure A3). Test005 produces alarms in all 149 scored frames, including normal frames. Avenue videos 03 (99.1%, Figure 2) and 16 (42.9%, Figure A4) illustrate favorable and unfavorable score ordering relative to the annotations. In Avenue video 16, repeated high scores occur outside the anomalous interval. IITB-Corridor video 000233 (Figure 1, bottom row) reaches the alarm threshold in 379 frames and has a within-video AUROC of 94.1%. At the peak frame, its largest deviations are in the mean horizontal velocity ($h_{5}$) and the median speed ($h_{1}$). IITB-Corridor video 000225 (Figure 3) illustrates false alarms in an entirely normal video.
